# PAQR3 modulates cholesterol homeostasis by anchoring Scap/SREBP complex to the Golgi apparatus

**DOI:** 10.1038/ncomms9100

**Published:** 2015-08-27

**Authors:** Daqian Xu, Zheng Wang, Yuxue Zhang, Wei Jiang, Yi Pan, Bao-Liang Song, Yan Chen

**Affiliations:** 1 Key Laboratory of Nutrition and Metabolism, Institute for Nutritional Sciences, Shanghai Institutes for Biological Sciences, Graduate School of the Chinese Academy of Sciences, Chinese Academy of Sciences, Shanghai 20031, China; 2 State Key Laboratory of Molecular Biology, Institute of Biochemistry and Cell Biology, Shanghai Institutes for Biological Sciences, Chinese Academy of Sciences, Shanghai 200031, China; 3 College of Life Sciences, the Institute for Advanced Studies, Wuhan University, Wuhan 430072, China

## Abstract

Cholesterol biosynthesis is regulated by transcription factors SREBPs and their escort protein Scap. On sterol depletion, Scap/SREBP complex is transported from endoplasmic reticulum (ER) to the Golgi apparatus where SREBP is activated. Under cholesterol sufficient condition, Insigs act as anchor proteins to retain Scap/SREBP in the ER. However, the anchor protein of Scap/SREBP in the Golgi is unknown. Here we report that a Golgi-localized membrane protein progestin and adipoQ receptors 3 (PAQR3) interacts with Scap and SREBP and tethers them to the Golgi. PAQR3 promotes Scap/SREBP complex formation, potentiates SREBP processing and enhances lipid synthesis. The mutually exclusive interaction between Scap and PAQR3 or Insig-1 is regulated by cholesterol level. PAQR3 knockdown in liver blunts SREBP pathway and decreases hepatic cholesterol content. Disrupting the interaction of PAQR3 with Scap/SREBP by a synthetic peptide inhibits SREBP processing and activation. Thus, PAQR3 regulates cholesterol homeostasis by anchoring Scap/SREBP to the Golgi and disruption of such function reduces cholesterol biosynthesis.

## Abstract (web summary)

Under conditions of sterol depletion, the Scap/SREBP complex is transported from the endoplasmic reticulum to the Golgi apparatus. Here Xu and Wang *et al.* show that the Golgi protein PAQR3 interacts with Scap and SREBP in a cholesterol regulated manner to help regulate sterol homeostasis.

## Graphical abstract

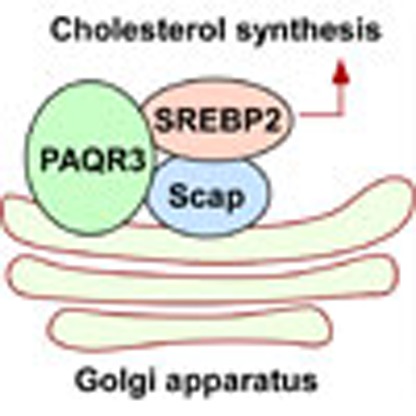

Cholesterol is a sterol lipid that plays important structural and functional roles in cellular membranous structures as it can provide rigidity to membrane fluidity. In addition, cholesterol and its metabolites, such as bile acids, oxysterols, certain vitamins, and steroid hormones, are essential for a variety of cellular functions. Dysregulation of cholesterol homeostasis is closely associated with the pathogenesis of many human diseases such as atherosclerosis, myocardial infarction and strokes. In mammals, cholesterol is acquired by both diet and *de novo* biosynthesis, while the latter being the major resource of cholesterol in mammals. Almost all mammalian cells are able to synthesize cholesterol *de novo*; however, the liver and small intestine are the two major organs that primarily provide cholesterol. Biosynthesis of cholesterol starts from acetyl coenzyme A and involves the cooperation of numerous enzymes in the mevalonate pathway^1^, in which HMG-CoA reductase (HMGCR) acts as the rate-limiting enzyme in cholesterol synthesis and the primary site of feedback regulation^2^,^3^,^4^.

The cellular cholesterol level is tightly regulated in a well-established feedback loop mainly contributed by Brown and Goldstein^5^. Three key proteins are involved in this feedback loop regulation: SREBPs (sterol regulatory element-binding proteins), Scap (SREBP cleavage-activating protein) and Insigs (insulin-induced genes). SREBPs are master regulators that control the transcription of critical genes involved in cholesterol synthesis, cholesterol uptake, as well as synthesis of fatty acid (FA) and triglyceride (TG)^5^. There exists three isoforms of SREBP in mammalian cells including SREBP-1a, SREBP-1c and SREBP-2. All three isoforms are closely related in sequence similarity and biological functions^6^,^7^. The major functional difference among these three SREBP isoforms is their preference for target genes^8^. SREBP-2 preferentially activates genes involved in cholesterol synthesis and uptake; SREBP-1c prefers to promote the transcription of genes related to FA and TG synthesis; while SREBP-1a is a potent activator for all SREBP-responsive genes^8^. The function of SREBPs is tightly modulated by an escort protein Scap and an endoplasmic reticulum (ER) anchor protein Insig during the feedback regulation of cholesterol synthesis. Scap protein contains eight transmembrane helices separated by hydrophilic loops, in which helices 2–6 constitute the sterol-sensing domain^9^,^10^. On sterol depletion, Scap/SREBP complex is transported from the ER to the Golgi apparatus (GA) via COPII-mediated vesicles^11^, mediated by binding of COPII coat protein Sec24 with a hexapeptide sequence MELADL located in the cytoplasmic loop between transmembrane helices 6 and 7 of Scap^12^. In the GA, SREBPs are cleaved sequentially by membrane-bound peptidases site-1 protease (S1P) and site-2 protease (S2P), resulting in release of an NH2-terminal transactivation domain that enters the nuclei to initiate transcription of genes involved in cholesterol and lipid synthesis^5^,^8^,^13^. When intracellular cholesterol builds up to the extent that the ER cholesterol exceeds 5% of total ER lipids^14^, cholesterol binds Scap and alters its confirmation, resulting in Insig binding with Scap. As a consequence, the MELADL motif can no longer interact with COPII coat proteins, and Scap and SREBP are retained in the ER^15^. Inasmuch as Insig functions as an ER anchor protein for Scap/SREBP complex, it is conceivable that there may exist a Golgi anchor protein that facilitates retention of the complex in the GA and participates in the regulation of cholesterol biosynthesis. This is what this study is trying to address.

PAQR3 is a member of the progestin and adipoQ receptors (PAQR) superfamily, and the prototype of this family is adiponectin receptors^16^. PAQR3 is a Golgi-anchored membrane protein containing seven transmembrane helices^17^,^18^. Previous studies revealed that PAQR3 functions as a tumour suppressor in regulating tumour cell proliferation and migration by negative regulation of Raf kinases and AKT pathway^17^,^19^,^20^,^21^,^22^,^23^. PAQR3 also modulates insulin sensitivity, energy metabolism, as well as obesity in mice partly via negative regulation of PI3K^24^,^25^. Intriguingly, the blood cholesterol and low density lipoprotein-cholesterol (LDL-C) levels of PAQR3-deleted mice fed with high fat diet were significantly reduced when compared with wild-type littermates^25^, initiating our study as reported here to uncover the novel functions of PAQR3 in the regulation of cholesterol biosynthesis.

Using a series of studies with cellular and mouse models, we demonstrate here that PAQR3 interacts with Scap/SREBP and tethers them to the Golgi complex, resulting in enhanced SREBP processing and cellular cholesterol level. Remarkably, PAQR3 and Insig can mutually exclusively compete for Scap interaction in a cholesterol-dependent manner. Importantly, knockdown of PAQR3 in mouse liver blunts SREBP pathway and decreases cholesterol synthesis. Disrupting the interaction of PAQR3 with Scap/SREBP by a synthetic peptide dramatically reduces SREBP processing and activation. Thus, PAQR3 plays a vital role in the regulation of SREBP pathway and cholesterol homeostasis *in vitro* and *in vivo* by acting as an anchor protein for Scap/SREBP complex in the GA.

## Results

### PAQR3 modulates SREBP-2 activation

**Figure 1:**
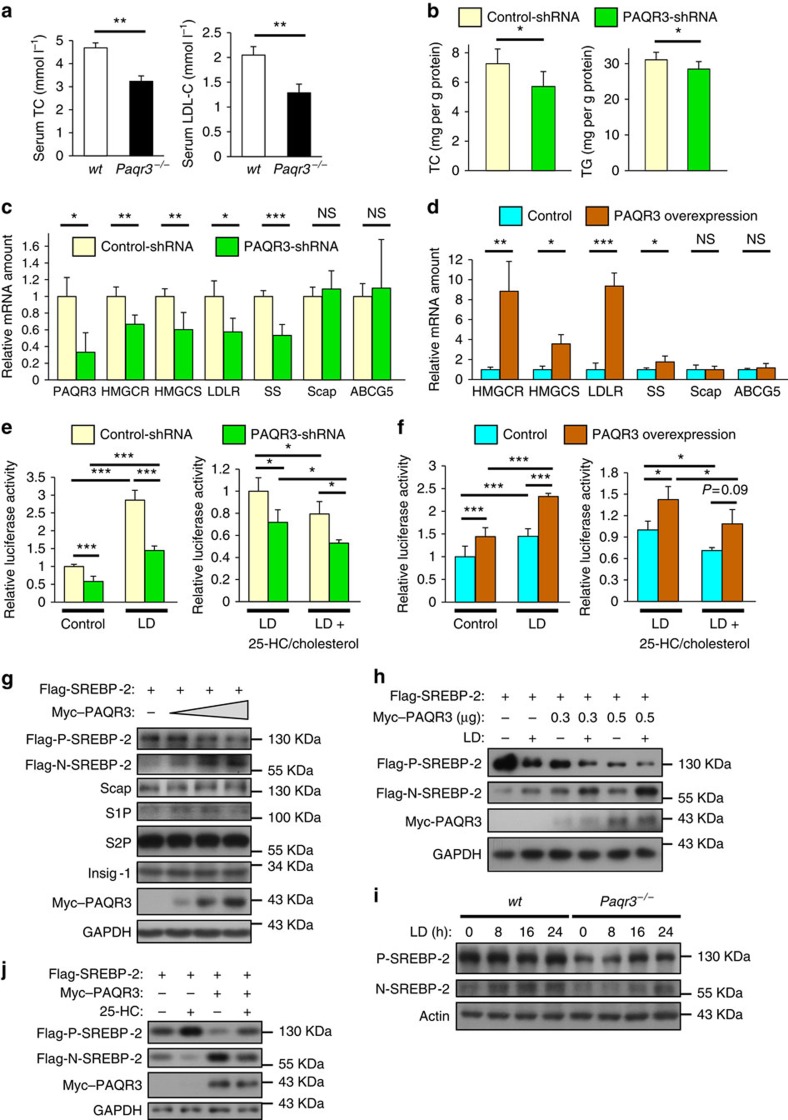
PAQR3 modulates SREBP activation and cholesterol biosynthesis. (**a**) Serum levels of total cholesterol (TC) and LDL-C measured in wild-type (*wt, n*=15) or PAQR3 deletion (*Paqr3*^*−/−*^, *n*=13) mice fed with high fat diet for 16 weeks.(**b**) Cellular TC and TG contents measured in primary hepatocyte infected with control-shRNA or PAQR3-shRNA adenoviruses. (**c**) Gene expression levels measured by reverse transcription–PCR (RT–PCR) in primary hepatocytes infected with control-shRNA or PAQR3-shRNA adenoviruses in normal medium. (**d**) Gene expression levels measured by RT–PCR in HepG2 cells transfected with control or PAQR3-overexpression plasmids in normal medium. (**e**,**f**) Huh7 cells were transiently transfected with the plasmids as indicated together with β-galactosidase and SRE-containing luciferase reporter. Luciferase activities were determined under lipid-loaded medium (control), lipid depletion medium (LD) with or without 25-HC/cholesterol replenishment after normalization to β-galactosidase activities. (**g**,**h**) Western blot analysis of CHO-7 cells transfected with the plasmids as indicated. Cells were cultured in normal medium (**g**) or LD medium (**h**). (**j**) Western blot analysis of CHO-7 cells transfected with the plasmids as indicated. Cells were cultured in LD medium for 16 h and then with or without 25-HC replenishment for 6 h. (**i**) Western blot analysis of primary hepatocyte from *wt* or *Paqr3*^*−/−*^ mice in normal medium or LD medium for different times as indicated. All bars show mean±s.d., *P<0.05, **P<0.01, ***P<0.001 by Student's *t*-test; NS, not significant. All experiments were repeated at least twice with similar results and representative data are shown.

The first clue that led us to investigate the potential role of PAQR3 in cholesterol synthesis was the discovery that the levels of blood cholesterol and LDL-C were significantly reduced by PAQR3 gene deletion in mice fed with high fat diet (Fig. 1a), as previously reported by our group^25^. PAQR3 deletion also led to slight reduction in blood cholesterol level but not LDL level with normal chow diet^25^. To confirm such findings, we investigated the effect of PAQR3 on cellular cholesterol and TG concentrations. Knockdown of PAQR3 significantly reduced cholesterol and TG levels in primary hepatocytes (Fig. 1b). Consistently, knockdown of PAQR3 in primary hepatocytes significantly reduced the mRNA levels of critical cholesterol-synthesizing and uptaking genes including *3-hydroxy-3-methylglutaryl-CoA reductase* (*HMGCR*), *HMGC synthase* (*HMGCS*), *LDL receptor* (*LDLR*) and *squalene synthase* (*SS*) (Fig. 1c). As negative controls, the mRNA levels of Scap and *ATP-binding cassette sub-family G* (*ABCG5*) were not altered by PAQR3 knockdown (Fig. 1c). On the other hand, overexpression of PAQR3 in HepG2 cells significantly elevated the expression of these genes (Fig. 1d). As all the genes mentioned above are target genes of SREBP-2, we examined the effect of PAQR3 on SREBP activation. We first analysed the effect of PAQR3 on the activity of SRE. PAQR3 downregulation could not only reduce SRE activity at basal level, but also alleviate lipid depletion (LD)-induced SRE activity (Fig. 1e, left panel). Meanwhile, PAQR3 knockdown was able to reduce SRE activity upon cholesterol replenishment (Fig. 1e, right panel). In contrast, overexpression of PAQR3 significantly elevated SRE activity at basal level and under LD condition (Fig. 1f).

We next explored whether PAQR3 exerts an effect on SREBP processing. Overexpression of PAQR3 in CHO-7 cells markedly increased proteolytic cleavage of SREBP-2 in a dose-dependent manner, shown as elevation of the mature nuclear form of SREBP-2 (N-SREBP-2) (Fig. 1g). Meanwhile, PAQR3 overexpression had no effect on other critical proteins involved in SREBP activation including Scap, Insig-1, S1P and S2P (Fig. 1g). Consistently, LD-mediated SREBP-2 processing was markedly enhanced by PAQR3 overexpression in CHO-7 cells (Fig. 1h). In contrast, LD-induced SREBP-2 cleavage was alleviated by PAQR3 deletion in primary hepatocytes (Fig. 1i). PAQR3 knockdown also profoundly reduced SREBP-2 processing in CHO-7 cells (Supplementary Fig. 1). As expected, addition of 25-hydroxycholesterol (25-HC) reduced SREBP-2 processing under LD condition, while PAQR3 overexpression could still enhance the cleavage of SREBP-2 (Fig. 1j and Supplementary Fig. 2a). Collectively, these results indicated that PAQR3 is involved in the regulation of cholesterol biosynthesis via modulation of SREBP-2 activity. It is noteworthy that PAQR3 not only affected SREBP-2 activation, but also had an effect on the activation of SREBP-1a and SREBP-1c (Supplementary Fig. 2b–e), which are mainly responsible for transcription of genes involved in synthesis of FA and TG. Nevertheless, we focused on the modulatory role of PAQR3 on cholesterol biosynthesis in this study.

### PAQR3 promotes localization of Scap/SREBP in the Golgi

There are two possibilities that may explain the enhancement of SREBP-2 processing by PAQR3: an increase in the enzyme activity of S1P/S2P or an increase in the localization of Scap/SREBP complex in the GA. To investigate the first possibility, we analysed whether PAQR3 had an effect on the enzyme activities of S1P and S2P by examining the cleavage of ATF-6, another proteolytic substrate of S1P and S2P^26^. We found that overexpression of PAQR3 could neither alter ATF-6 processing in normal medium nor on tunicamycin treatment (Supplementary Fig. 2f,g), indicating that the observed alteration in SREBP processing by PAQR3 is not likely caused by changes in S1P and S2P activities.

**Figure 2:**
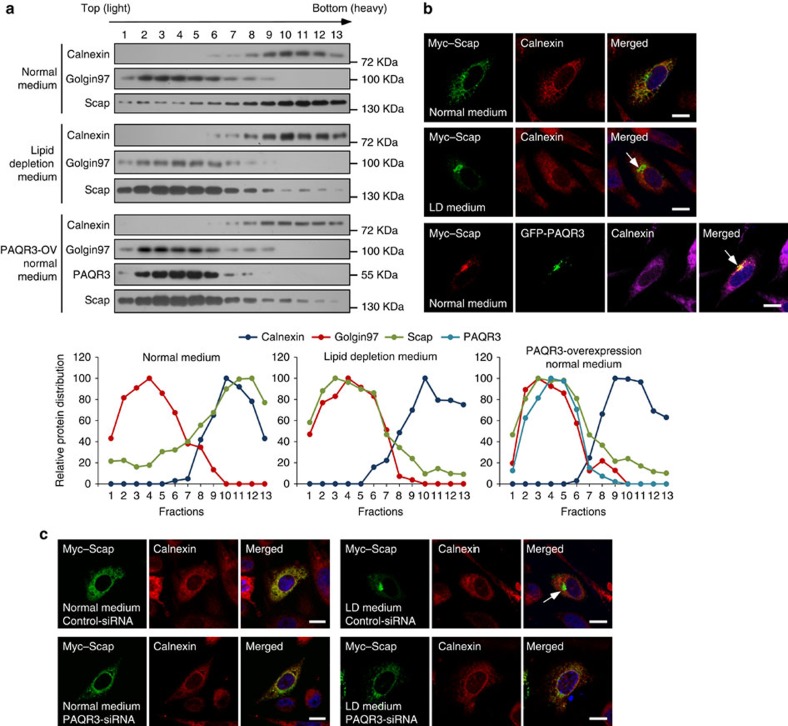
PAQR3 tethers Scap to the Golgi apparatus. (**a**) Analysis of Scap and PAQR3 subcellular localization by fractionation. Myc-tagged Scap (for all three groups) and GFP-fused PAQR3 (for the last group) were transfected into sterol-sensitive and Scap-deficient SRD-13A cells. At 36 h after the transfection, the cells were switched to normal medium or lipid depletion medium for 90 min, and then subjected to homogenization and cell fractionation by gradient centrifugation. The relative distribution of each protein in different fractions is shown in the lower panels after densitometry analysis of the blots. (**b**) PAQR3 overexpression promotes Golgi localization of Scap. SRD-13A cells were transiently transfected with the plasmids as in **a** and used in immunofluorescence staining and confocal analysis. The arrows indicate apparent localization of Scap in the Golgi. (**c**) Knockdown of PAQR3 reduces lipid depletion-induced Golgi localization of Scap. SRD-13A cells were transfected either with control siRNA or PAQR3 siRNA. At 24 h after transfection, the cells were transfected with Myc-tagged Scap and cultured for 24 h. Then the cells were subjected to normal culture medium or lipid depletion medium before immunofluorescence staining and confocal analysis. The arrow indicates LD-induced localization of Scap in the Golgi. Scale bar,10 μm. All experiments were repeated at least twice with similar results and representative data are shown.

As PAQR3 is a Golgi-localized membrane protein^17^,^18^, we next explored the possibility that PAQR3 might alter subcellular localization of Scap and SREBP. We employed a biochemical strategy to analyse ER and GA membrane fractions by gradient centrifugation using a method as previously reported^27^. In sterol-sensitive and Scap-deficient SRD-13A cells, ectopically expressed Scap was mainly present in the ER fractions in normal medium (Fig. 2a). On LD, the majority of Scap was shifted to the Golgi fractions in SRD-13A cells (Fig. 2a), consistent with previous reports^11^. Intriguingly, PAQR3 overexpression caused the majority of Scap to retain in the Golgi fractions in normal medium (Fig. 2a). As a complement experiment, we used immunostaining to confirm the change of Scap localization in SRD-13A cells (Fig. 2b). Consistent with our fractionation results and previous studies^11^, Scap was mainly co-localized with an ER marker calnexin in normal medium. LD led to the majority of Scap being mobilized to the GA. Overexpression of PAQR3 itself could shift the localization of Scap from ER to the GA in normal medium. We also analysed the effect of PAQR3 knockdown on the localization of expressed Scap in SRD-13A cells. In normal medium, the majority of Scap resided in the ER (Fig. 2c). As expected, LD induced the majority of Scap to relocate to the GA after control-siRNA transfection (Fig. 2c). However, PAQR3 knockdown markedly abrogated LD-induced Golgi localization of Scap (Fig. 2c). These data, therefore indicated that PAQR3 could promote anchoring of Scap to the GA.

We also investigated the effect of PAQR3 overexpression on the localization of Scap and SREBP in Hela cells. Under normal culture condition, exogenously expressed Scap was distributed mainly in ER and hardly in the GA in normal medium (Supplementary Fig. 3a,b). However, when co-expressed with PAQR3, almost all Scap proteins were localized in the GA (Supplementary Fig. 3c,j). Similarly, SREBP-2 was mainly localized in the ER but not in the GA (Supplementary Fig. 3d,e). However, when SREBP-2 was co-expressed with PAQR3, the majority of SREBP-2 was localized in the GA together with PAQR3 (Supplementary Fig. 2f,j). In contrast, overexpression of PAQR3 did not alter the localization of Insig-1, which stayed in the ER (Supplementary Fig. 3d–i). Therefore, these data provided further evidence that PAQR3 is able to alter the cellular localization of Scap and SREBP-2 by tethering them to the GA (Supplementary Fig. 3j).

### PAQR3 enhances Scap/SREBP complex formation

**Figure 3:**
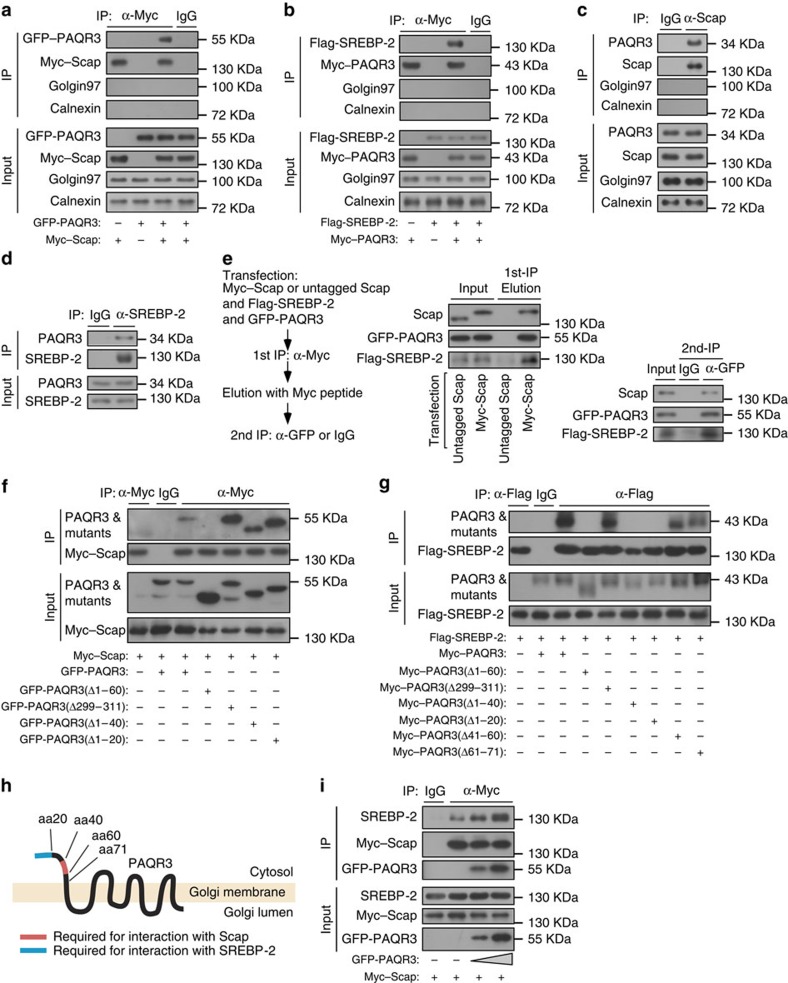
PAQR3 interacts with SREBP-2 and Scap via distinct structural motifs. (**a**,**b**) Interaction of PAQR3 with Scap and SREBP-2. HEK293T cells were transiently transfected with the plasmids as indicated. The cell lysate was used in immunoblotting (IB) and immunoprecipitation (IP) with the antibodies as indicated. (**c**,**d**) Interaction of endogenous PAQR3 with endogenous Scap and SREBP-2 in HEK293T cells. The cell lysate was used in IB and IP using the antibodies as indicated. (**e**) A two-step co-immunoprecipitation assay to determine the ternary complex containing PAQR3, Scap and SREBP-2. The procedures of the two-step co-immunoprecipitation are outlined in the left. HEK293T cells were transfected with the plasmids as indicated and used in the immunoprecipitation. (**f**,**g**) Determination of PAQR3 domains required for its interaction with Scap and SREBP-2. Different PAQR3 deletion constructs were co-transfected with Myc-tagged Scap (**f**) or Flag-tagged SREBP-2 (**g**) into HEK293T cells. The cells were then subjected to lysis and IP, followed by IB with different antibodies as indicated. (**h**) A schematic diagram depicts critical domains of PAQR3 involved in the interaction with Scap and SREBP-2, respectively. (**i**) PAQR3 promotes Scap and SREBP-2 interaction. HEK293T cells were transiently transfected with the plasmids as indicated and the cell lysate was used in IB and IP with the antibodies as indicated. All experiments were repeated three times with similar results and representative data are shown.

The alteration of subcellular compartmentalization of Scap and SREBP-2 by PAQR3 prompted us to investigate the potential interaction of PAQR3 with these two proteins. We used co-immunoprecipitation assays to determine whether PAQR3 could form a complex with Scap and SREBP-2. When both PAQR3 and Scap were overexpressed in HEK293T cells, immunoprecipitation of Scap could bring down PAQR3, but not ER and GA markers calnexin and golgin-97 (Fig. 3a). Similarly, when both PAQR3 and SREBP-2 were overexpressed, PAQR3 co-precipitated with SREBP-2 (Fig. 3b). Importantly, endogenous PAQR3 was able to interact with endogenous Scap or endogenous SREBP-2 by the co-immunoprecipitation assays (Fig. 3c,d). Furthermore, we found that PAQR3, Scap and SREBP-2 could form a ternary complex by a two-step co-immunoprecipitation assay (Fig. 3e). To further confirm the binding specificity of PAQR3 and Scap/SREBP, we examined the interaction of PAQR3 with other Golgi-related proteins including S1P/S2P, the Golgi resident proteins; VSVG, a protein that cycles between the ER and Golgi; and albumin, a secretory protein through the Golgi. We found that PAQR3 could not interact with anyone of these proteins (Supplementary Fig. 4).

To characterize the structural motifs of PAQR3 involved in the interaction with Scap and SREBP-2, we employed a series of NH_2_-terminal deletion mutants of PAQR3, as our previous studies have identified the NH_2_-terminal 71 amino acid residues facing the cytosol as critical for the functionality of PAQR3 (refs 17, 18). By co-immunoprecipitation assays, it was evident that deletion of amino acid residues 1–60, but not 1–20 nor 1–40 of PAQR3 lost the ability to interact with Scap (Fig. 3f), indicating that the amino acid residues 41–60 are required for binding of PAQR3 with Scap. On the other hand, deletions of amino acid residues 1–60, 1–40 and 1–20 lost the ability of PAQR3 to interact with SREBP-2 (Fig. 3g), suggesting that the first 20 amino acid residues of PAQR3 are implicated in SREBP-2 binding. Collectively, these data pinpointed that PAQR3 forms a ternary complex with Scap and SREBP-2 via distinct motifs (Fig. 3h). Based on the structural investigation, we postulated that PAQR3 was able to promote the interaction of Scap with SREBP-2 via simultaneous binding with the two proteins. Intriguingly, overexpression of PAQR3 could dose-dependently promote the complex formation between Scap and SREBP-2 (Fig. 3i). Therefore, PAQR3 not only interacts with both Scap and SREBP, but also promotes Scap/SREBP complex formation.

### Cholesterol modulates Scap interaction with PAQR3 or Insig

**Figure 4:**
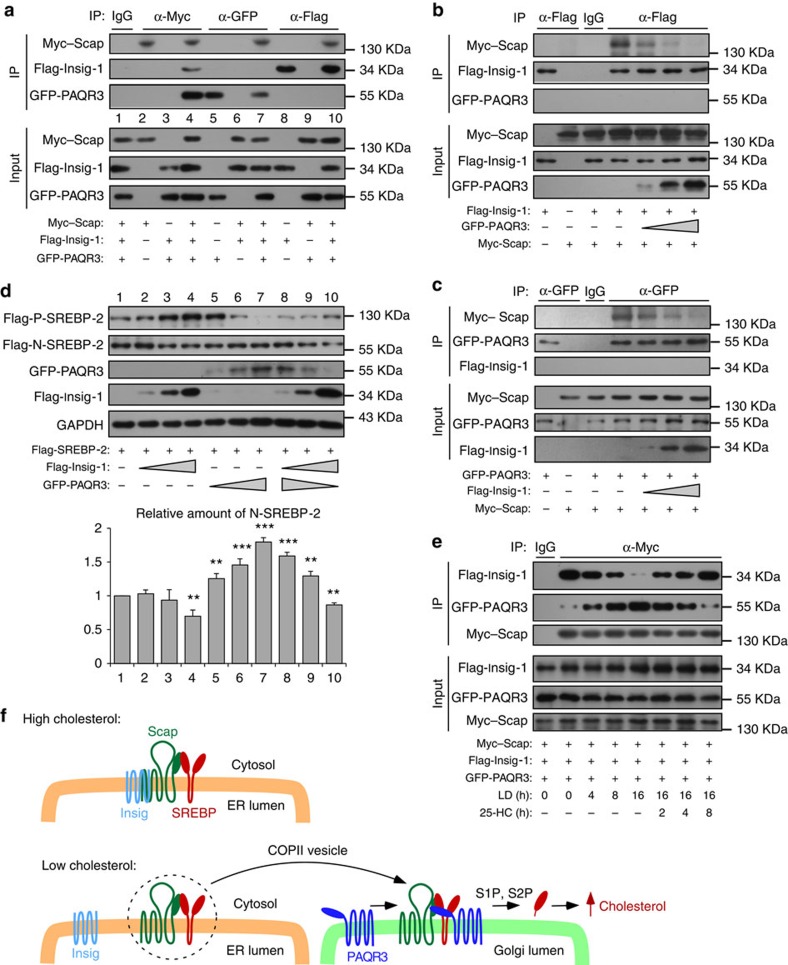
Mutually exclusive interaction of Scap with PAQR3 or Insig-1 in the control of cholesterol homeostasis. (**a**) Mutually exclusive interaction of Scap with PAQR3 or Insig-1. HEK293T cells were transfected with the plasmids as indicated and the cell lysate was used in immunoblotting (IB) and immunoprecipitation (IP) with the antibodies as indicated. (**b**,**c**) Mutually exclusive competition between PAQR3 and Insig-1 for Scap binding. HEK293T cells were transfected with plasmids as indicated, followed by IB and IP. (**d**) The relative levels of PAQR3 and Insig-1 determine SREBP-2 activation. CHO-7 cells were transfected with the plasmids as indicated and the cell lysate was used in IB. The relative ratios of N-SREBP-2 versus P-SREBP-2 were obtained by densitometric analysis (shown in the lower penal, **P<0.01, ***P<0.001 by Student's *t*-test). (**e**) The interaction of Scap with PAQR3 or Insig-1 is regulated by cholesterol. HEK293T cells were transfected with the plasmids as indicated and subjected to normal medium, LD medium with or without 25-HC replenishment for different time as indicated, followed by IB and IP assays. All experiments were repeated three times with similar results and representative data are shown.(**f**) A model depicts the functional role of PAQR3 in regulating cholesterol biosynthesis. When cellular cholesterol level is high, Scap/SREBP complex is retained in the ER by Insig, resulting in inactivation of SREBP. When cholesterol level is low, Insig is separated from Scap/SREBP complex which is then transported to the GA via COPII vesicles. PAQR3 facilitates anchoring of Scap/SREBP complex in the GA and promotes SREBP activation. What is not illustrated in the model is that this process is regulated by cholesterol. Low cholesterol induces degradation of Insig-1 while elevates PAQR3 expression, favouring retention of Scap/SREBP complex in the GA.

As both Insig and PAQR3 can interact with Scap in different subcellular compartments, we hypothesized that the interaction of Scap with PAQR3 or Insig should control the balance of cholesterol homeostasis. Insig promotes retention of Scap in the ER and inactivation of SREBP, while PAQR3 facilitates retention of Scap in the GA and activation of SREBP. In exploring this hypothesis, we first confirmed that the interactions of PAQR3 and Insig with Scap were mutually exclusive. We performed a co-immunoprecipitation assay in which all three proteins, that is, PAQR3, Insig-1 and Scap, were co-expressed (Fig. 4a). Immunoprecipitation of Scap could bring down both PAQR3 and Insig-1 (Fig. 4a, lanes 2–4). Immunoprecipitation of PAQR3 could only bring down Scap but not Insig-1 (Fig. 4a, lanes 5–7). Likewise, immunoprecipitation of Insig-1 could only bring down Scap but not PAQR3 (Fig. 4a, lanes 8–10).

Since PAQR3 and Insig-1 are not present in the same protein complex, we next analysed whether the interaction of Insig-1 with Scap was modulated by the expression level of PAQR3. By a co-immunoprecipitation assay, we found that overexpression of PAQR3 was able to reduce Insig/Scap interaction in a dose-dependent manner (Fig. 4b). On the other hand, overexpression of Insig-1 was able to lessen the interaction of PAQR3 with Scap in a dose-dependent manner (Fig. 4c). These results, therefore, indicated that PAQR3 and Insig can mutually exclusively compete for Scap interaction, leading to either activation of SREBP by anchoring it to the GA (via PAQR3) or inactivation of SREBP by anchoring it to the ER (via Insig). Such presumptions were supported by our findings that increasing expression of Insig-1 reduced SREBP-2 processing, while increasing expression of PAQR3 elevated SREBP-2 processing (Fig. 4d, lanes 2–7). Furthermore, the magnitude of SREBP-2 activation was determined by the relative amount of Insig-1 versus that of PAQR3 (Fig. 4d, lanes 8–10).

What is the significance of the mutually exclusive activities of PAQR3 versus Insig in Scap interaction? As the transport of Scap/SREBP complex between ER and GA is regulated by cellular cholesterol concentration, we postulated that Insig would favour Scap/SREBP inactivation under cholesterol-rich conditions, while PAQR3 would favour Scap/SREBP activation under cholesterol-depletion conditions. We designed an experiment to address this hypothesis. We found that LD, a condition that releases Scap from Insig interaction, could markedly enhance the interaction of Scap with PAQR3 (Fig. 4e and Supplementary Fig. 5). On the other hand, addition of 25-HC, a sterol that promotes Insig/Scap interaction, reduced Scap interaction with PAQR3 (Fig. 4e). Interestingly, the mRNA level of PAQR3 was elevated by LD (Supplementary Fig. 6a). The PAQR3 promoter activity could be enhanced by SREBP-2 in both normal and LD media (Supplementary Fig. 6b). Therefore, we propose that PAQR3 plays a pivotal role in modulating cholesterol homeostasis under cholesterol-depletion condition when cholesterol biosynthesis machinery is required to be executed (Fig. 4f). Under low cholesterol condition, Insig is removed from the Scap/SREBP complex and undergoes rapid degradation. The Scap/SREBP is transported from ER to the Golgi, in which PAQR3 facilitates the retention of the complex in the GA. LD-induced expression of PAQR3 further enhances this process. As a consequence, SREBP is cleaved by GA-localized proteases and cholesterol biosynthesis is initiated. On the other hand, high level of cholesterol favors Scap/SREBP interaction with Insig, leading to retention of the complex in the ER and inactivation of SREBP. The reduced degradation of Insig and decreased expression of PAQR3 under high cholesterol condition would further facilitate this process.

### PAQR3 modulates SREBP activity in fasting and feeding cycle

**Figure 5:**
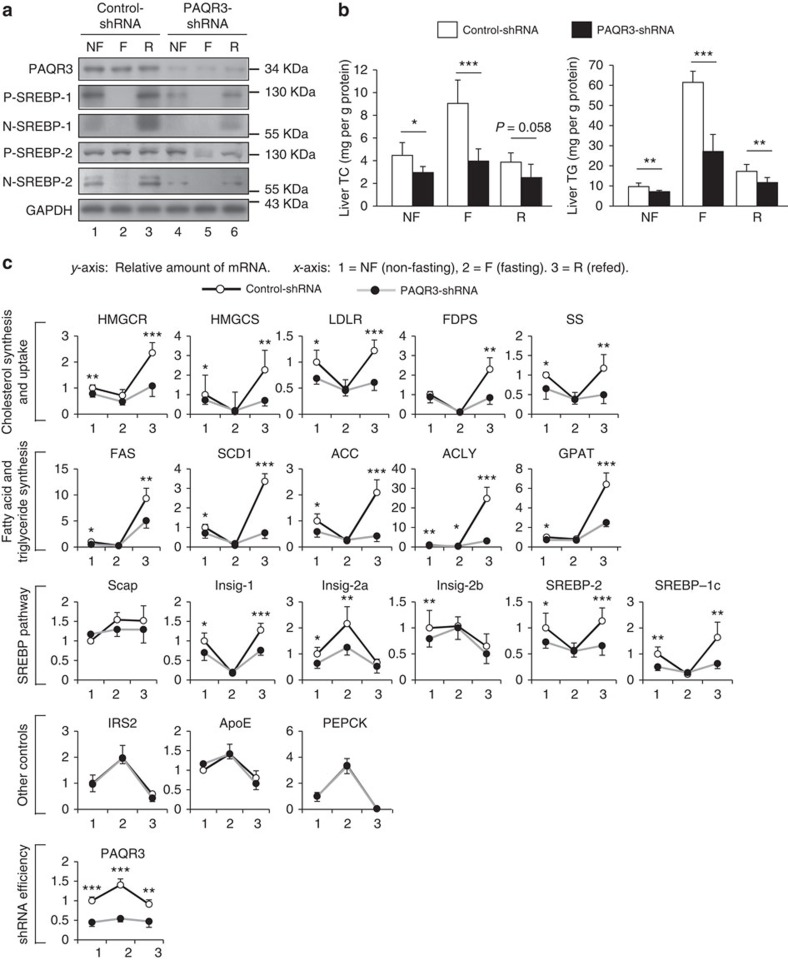
PAQR3 modulates SREBP activity and lipid synthesis of the liver on refeeding. Eight-week-old male C57BL/6J mice were injected with control or PAQR3 knockdown adenovirus i.v. Seven days after injection, the mice (*n*=6 per group) were subjected to fasting and refeeding. The non-fasted group (NF) was fed a chow diet *ad libitum*, the fasted group (F) was fasted for 24 h, and the refed group (R) was fasted for 24 h and then refed a high-carbohydrate/low-fat diet for 12 h. (**a**) Western blot analysis of the mouse livers. (**b**) Total cholesterol (TC) and triglyceride (TG) contents in the livers. (**c**) Gene expression profiles in the mouse liver measured by RT–PCR. The same experiment was repeated twice with similar results. All data show mean±s.d., *P<0.05, **P<0.01, ***P<0.001 by Student's *t*-test.

The aforementioned studies revealed the significance of PAQR3 in regulating SREBP activities at the cellular level. It was previously reported that the processing and activation of SREBP was regulated by fasting and feeding^28^. To examine the physiological functions of PAQR3, we used a model in which PAQR3 expression was downregulated by a PAQR3-specific shRNA^20^. Recombined adenoviruses that contained control-shRNA or PAQR3-shRNA were injected into C57BL/6J mice via tail vein and such injection would target expression of endogenous genes exclusively in the liver^29^. We first analysed whether PAQR3 knockdown could modulate SREBP activation. Consistent with a previous report^28^, fasting reduced the precursor and nuclear forms of SREBPs in the control mice (Fig. 5a, lane 2), and refeeding induced an ‘overshoot' in SREBP activation (Fig. 5a, lane 3). In contrast, the ‘overshoot' of SREBP activation upon refeeding was markedly reduced by PAQR3 knockdown (Fig. 5a, lane 6 compared to lane 3), indicating that PAQR3 did have an effect in modulating SREBP activation *in vivo*. Consistently, we found that the levels of liver cholesterol and TG were also reduced by PAQR3 knockdown upon refeeding (Fig. 5b).

As previously reported^28^,^30^, fasting and refeeding caused a series of changes in gene expression at the mRNA level, including genes in SREBP pathway, cholesterol synthesis and uptake, as well as FA and TG synthesis. We analysed the mRNA levels of these genes in our mouse model (Fig. 5c). Under non-fasting condition, the SREBP-target genes including *HMGCR, HMGCS, LDLR, FDPS* (farnesyl diphosphate synthase), *SS, FAS* (FA synthase), *SCD-1* (stearoyl CoA desaturase-1), *ACC* (acetyl CoA carboxylase), *ACLY* (ATP citrate lyase), *GPAT* (glycerol-3-phosphate acyltransferase), *SREBP-1c*, *SREBP-2*, and *Insig-1* were all lower in PAQR3-shRNA group than in the control mice. Fasting caused the repression of these genes in both control and PAQR3-shRNA groups. Due to the ‘overshoot' of SREBP activation under refeeding condition, refeeding caused marked increases in all of the genes involved in the biosynthesis of cholesterol, FA and TG. However, such upregulation of these genes upon refeeding was robustly blunted by PAQR3 knockdown (Fig. 5c). It is noteworthy that the observed change of Insig-2a during the fasting-refeeding cycle was reciprocal of Insig-1, consistent with a previous report^28^. Nevertheless, PAQR3 also modulated the expression of Insig-2a mainly during fasting (Fig. 5c). As a control for the whole experiment, a few other genes that are not targeted by SREBPs, including *Scap*, *IRS2* (insulin receptor substrate-2), *ApoE* (apolipo-protein E), and *PEPCK* (phosphoenolpyruvate carboxykinase) were not affected by PAQR3 knockdown. Lastly, as expected, the PAQR3 expression level was significantly reduced by PAQR3-shRNA (Fig. 5c). Collectively, these data indicated that PAQR3 has a profound effect on modulating SREBP activity and the expression of SREBP downstream genes during fasting-refeeding cycle.

### PAQR3 regulates SREBP activity in various cholesterol diets

**Figure 6:**
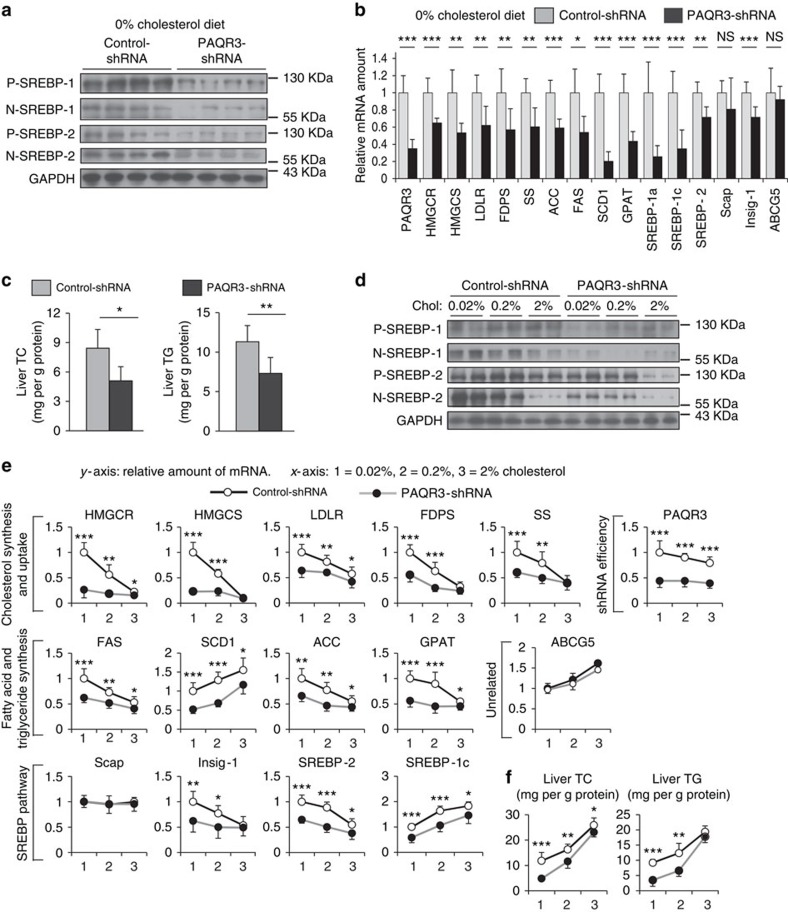
PAQR3 modulates SREBP activity and lipid biosynthesis mainly at low dietary cholesterol levels. Seven-week-old male C57BL/6J mice were injected with control or PAQR3-shRNA adenovirus i.v. Seven days after injection, the mice (*n*=7 per group) were subjected to diets containing different cholesterol levels *ad libitum* for 3 days, and then killed for analysis. (**a**–**c**) Western blotting analysis, gene expression levels as measured by RT–PCR and lipid measurement in the livers of mice fed with a diet containing 0% cholesterol. (**d**–**f**) Western blotting analysis, mRNA and lipid levels in the livers of mice fed with diets of varying cholesterol concentrations. All data show mean±s.d., *P<0.05, **P<0.01, ***P<0.001 by Student's *t*-test, NS, not significant. All experiments were repeated at least twice with similar results and representative data are shown.

Dietary absorption and biosynthesis are two sources for cholesterol acquisition in mammals. We next analysed whether PAQR3 has an effect on lipid synthesis in different diets with various cholesterol levels. At first, we analysed the effect of PAQR3 downregulation on SREBP activation in the livers of mice fed with a diet of 0% cholesterol. Compared with the control group, both the precursor and nuclear forms of SREBP-1 and SREBP-2 were reduced by adenovirus-induced PAQR3 knockdown in the liver (Fig. 6a). Consequently, PAQR3 knockdown in liver not only downregulated the expression of the genes regulated by SREBPs including *HMGCR*, *HMGCS*, *LDLR*, *FDPS*, *SS*, *ACC*, *FAS*, *SCD-1* and *GPAT*, but also ameliorated gene expression in SREBP pathway, including *SREBP-1a*, *SREBP-1c*, *SREBP-2* and *Insig-1*,without change in *Scap* expression (Fig. 6b). Analysis for hepatic lipid levels revealed a profound reduction in total cholesterol (TC) and TG contents by PAQR3 knockdown in 0% cholesterol diet (Fig. 6c). On the other hand, overexpression of PAQR3 in the liver was able to augment SREBP processing and the expression of SREBP-target genes in normal chow and 0% cholesterol diet (Supplementary Fig. 7a–d). Besides, TC and TG contents in the liver were significantly elevated by PAQR3 overexpression (Supplementary Fig. 7e).

We next investigated the effect of PAQR3 on SREBP activity in the context of various cholesterol levels in the diet. The mice were fed with chow diet supplemented with 0.02, 0.2 and 2% of cholesterol. As expected, activations of SREBP-1 and SREBP-2 were very prominent under low cholesterol conditions and diminished by high cholesterol levels in the diet (Fig. 6d). PAQR3 knockdown greatly reduced SREBP activation in low cholesterol diets (Fig. 6d). However, at 2% cholesterol level, PAQR3 knockdown had minimal effect on SREBP activation (Fig. 6d). Consistently, knockdown of PAQR3 prominently reduced the mRNA levels of SREBP-target genes at low cholesterol levels, but not at the 2% cholesterol level (Fig. 6e). In addition, the effects of PAQR3 knockdown on liver TC and TG levels were maximal at low cholesterol concentrations (Fig. 6f). Collectively, these data indicated that PAQR3 exerts its effect on lipid biosynthesis mainly under a low cholesterol condition in which cholesterol synthesis is required.

### A peptide inhibits SREBP activation and lipid synthesis

**Figure 7:**
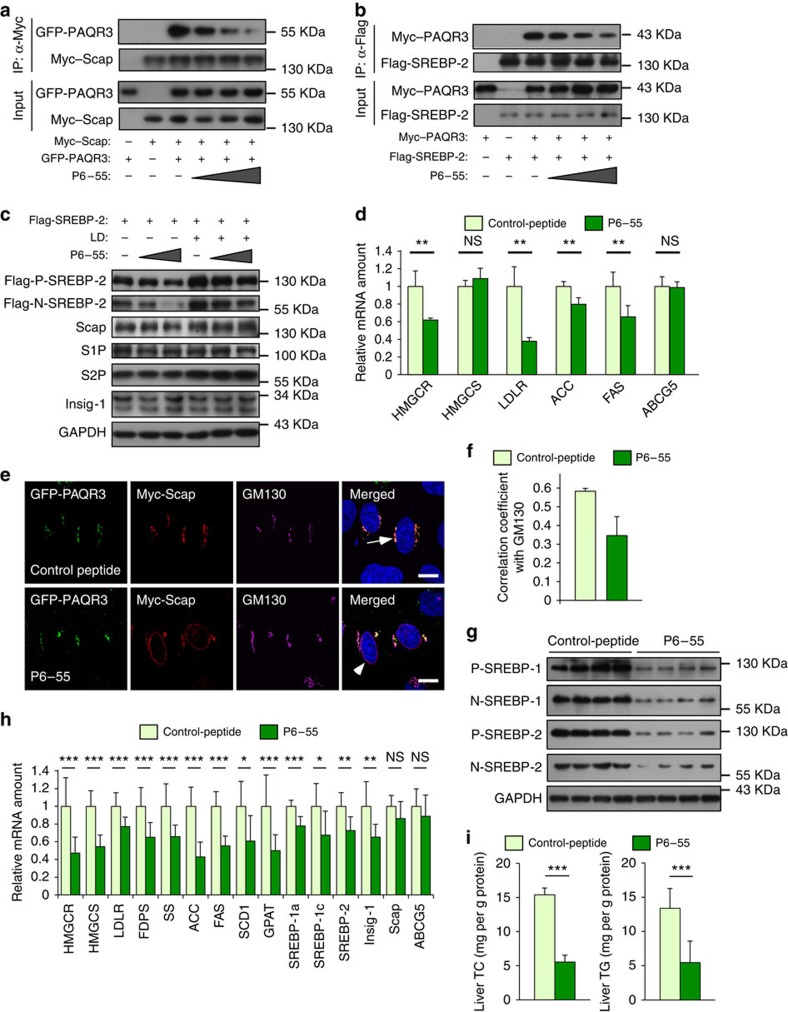
Disruption of PAQR3 interaction with Scap/SREBP by a synthetic peptide effectively blunts SREBP activity and lipid biosynthesis in the liver. (**a**,**b**) A synthetic peptide P6–55 blocks the interaction of PAQR3 with Scap and SREBP-2. HEK293T cells were transfected with the plasmids as indicated. At 24 h after transfection, the cells were treated with P6–55 (1, 4 or 20 ng μl^−1^) for 12 h and the cell lysate was used for immunoblotting (IB) and immunoprecipitation (IP) using the indicated antibodies. (**c**) P6–55 reduces SREBP activation. CHO-7 cell was transfected with Flag-tagged SREBP-2. At 6 h after transfection, the cells were treated with P6–55 (4 or 20 ng μl^−1^) in normal medium or LD medium for 16 h, and the cell lysate was used in western blotting with the indicated antibodies. (**d**) P6–55 decreases lipid-synthesizing gene expression in cells. CHO-7 cells were treated with control peptide or P6–55 (20 ng μl^−1^) for 96 h and then used in RT–PCR. (**e**,**f**) P6–55 alleviates PAQR3-mediated Golgi localization of Scap. HeLa cells were transiently transfected with PAQR3 and Scap. After culturing in normal medium for 48 h, the cells were treated with 20 ng μl^−1^ of control peptide or P6–55 for 12 h before immunofluorescence staining and confocal analysis. The nuclei were stained with Hoechst 33342 (only shown in the merged images). The arrows denote co-localization of PAQR3 with Scap in the GA. The arrowheads indicate apparent loss of co-localization of PAQR3 with Scap in the GA. Quantitation of the co-localization of the proteins in the GA is shown in **f**. (**g**–**i**) P6–55 shuts down SREBP activation and cholesterol synthesis in the liver. Western blotting analysis, gene expression analysis and lipid levels in the livers of mice (*n*=7 per group) treated with a control peptide or P6–55 (500 μg kg^−1^ per day) for 14 days. All bars show mean±s.d., *P<0.05, **P<0.01, ***P<0.001 by Student's *t*-test; NS, not significant. Scale bar, 10 μm. All experiments were repeated at least twice with similar results and representative data are shown.

As a proof-of-concept, we investigated whether disruption of the interaction of PAQR3 with Scap/SREBP can serve as a therapeutic means to lower cholesterol biosynthesis. Based on the investigation on the structural motifs involved in the interaction of PAQR3 with Scap and SREBP-2 (Fig. 3), we designed a synthetic peptide (P6–55) that covered the amino acid residues 6–55 in the NH_2_ terminus of PAQR3 protein and such region is implicated in the interaction of PAQR3 with both Scap and SREBP. A TAT sequence (RKKRRQRRR) was added in the NH_2_ terminus of the peptide to facilitate membrane penetrance^31^. As expected, P6–55 was able to reduce PAQR3/Scap interaction (Fig. 7a) and PAQR3/SREBP-2 interaction (Fig. 7b) in a dose-dependent manner. P6–55 was also able to inhibit SREBP processing in CHO-7 cells either in normal medium or in LD medium, but the protein levels of Scap, S1P, S2P and Insig-1 were not altered (Fig. 7c). As a control, we found that the control peptide containing only the TAT sequence had no effect on the interaction of PAQR3 with Scap/SREBP, and the activation of SREBPs (Supplementary Fig. 8).

P6–55 also significantly inhibited the expression of most SREBP-target genes such as *HMGCR*, *HMGCS*, *LDLR*, *ACC* and *FAS* at the cellular level (Fig. 7d). We also analysed the effect of P6–55 on subcellular localization of Scap. Overexpression of PAQR3 was able to tether most Scap to the GA; however, P6–55 was able to significantly reduce PAQR3-facilitated GA localization of Scap (Fig. 7e,f).

We next examined the *in vivo* functions of P6–55. C57BL/6 mice were given 500 μg kg^−1^ of control peptide or P6–55 daily by i.p. injection for 14 days. P6–55 administration dramatically reduced SREBP processing and the expression of SREBP-target genes in the liver (Fig. 7g,h). Finally, hepatic levels of TC and TG were significantly reduced by P6–55 (Fig. 7i). P6–55 also slightly lowered blood TG and cholesterol levels in the mice (from 0.42±0.08 to 0.35±0.05 mM, *P*<0.05 by Student's *t*-test). Collectively, these data indicated that disruption of PAQR3 interaction with Scap/SREBP is able to effectively blunt SREBP activation and lipid biosynthesis both *in vitro* and *in vivo*.

We also used these peptides to investigate the potential mechanism of PAQR3 on the traffic of Scap in SRD-13A cells (Supplementary Fig. 9a). While the cells stayed in the LD medium, we added a control peptide or P6–55 peptide that blocks the interaction of PAQR3 with Scap/SREBP. We found that the control peptide had no effect on Golgi localization of Scap. However, the P6–55 peptide markedly reduced Golgi localization of Scap. These data, therefore, supported a model that if Scap arrives in the Golgi and does not encounter PAQR3 it would not stay long in the Golgi and would traffic back to the ER (Supplementary Fig. 9b), further supporting the Golgi ‘anchoring' function of PAQR3.

## Discussion

Through a series of experiments at the biochemical, cellular and animal levels, we established that PAQR3 is an indispensable player that anchors Scap/SREBP to the GA and controls cholesterol homeostasis. The expression level of PAQR3 is crucial for the activities of SREBP: overexpression of PAQR3 stimulates SREBP activation, while downregulation of PAQR3 reduces SREBP activation (Fig. 1). Consequently, the expression of all the SREBP-target genes is regulated by PAQR3. Cell fractionation experiment indicates that PAQR3 tethers Scap to the GA, similar to LD-induced Golgi localization of Scap in SRD-13A cells (Fig. 2). Overexpression of PAQR3 also elevates Golgi localization of Scap and SREBP-2 but not Insig-1 (Supplementary Fig. 3). Furthermore, PAQR3 interacts with Scap and SREBP-2 via distinct structural motifs and promotes Scap/SREBP complex formation (Fig. 3). Importantly, PAQR3 and Insig-1 mutually exclusively interact with Scap in a manner regulated by cellular cholesterol levels, contributing to the balance of cholesterol homeostasis by altering the subcellular compartmentalization of Scap/SREBP complex (Fig. 4). Therefore, PAQR3 is actively involved in cholesterol biosynthesis by facilitating Scap/SREBP retention in the GA and promotes SREBP activation under cholesterol-depletion condition (Fig. 4f). As PAQR3 expression is stimulated by LD (Supplementary Fig. 6), it is likely that the LD-stimulated PAQR3 expression would form a positive feedback to accelerate SREBP activation in the GA by enhancing Scap/SREBP localization in the GA.

Our functional studies using various mouse models have further corroborated the importance of PAQR3 in lipid biosynthesis. First, we found that PAQR3 knockdown markedly reduces the refeeding-induced ‘overshoot' of SREBP activation (Fig. 5). These observations were quite similar to what were observed in liver specific Insig-1 transgenic mice^28^ and gp78 knockout mice^32^, further supporting our model that PAQR3 has an opposite function versus Insig in controlling lipid synthesis. Second, SREBP processing, liver cholesterol and TG concentrations, as well as the expression of SREBP-target genes in the liver are reduced by PAQR3 downregulation, especially under low cholesterol condition in which *de novo* lipid synthesis is initiated (Fig. 6). It is noteworthy that the total levels of SREBP proteins are also reduced by PAQR3 knockdown (Figs 5 and 6). This observation is likely due to the fact that the transcription of SREBP is under the control of SREBP itself in a positive feedback. Reduction of SREBP activation by PAQR3 knockdown can blunt this positive feedback, leading to reduction of SREBP expression.

Intriguingly, we found that the unique function of PAQR3 on cholesterol homeostasis can be exploited to lower lipid synthesis in the liver. As a proof-of-concept, we designed a synthetic peptide (P6–55) to block the interaction of PAQR3 with Scap/SREBP and investigated its effect on lipid homeostasis (Fig. 7). At the cellular level, P6–55 is able to disrupt the complex formation of PAQR3 with Scap and SREBP, block GA-anchoring of Scap and SREBP and blunt LD-induced activation of SREBP-2. At the animal level, P6–55 markedly decreases cholesterol and TG levels in the liver by reducing SREBP activation and the expression of its target genes. Taken together, our study is similar to the findings that small chemicals, such as fatostatin and betulin, that block ER-Golgi transport of SREBPs, are effective in inhibiting SREBP activation and reducing lipogenesis^33^,^34^. Therefore, disruption of the functionality of PAQR3 interaction with Scap/SREBP appears to be a potentially promising strategy for lowering cholesterol biosynthesis in the liver.

Even though we have provided convincing evidence that a Golgi anchor protein is of great importance in modulating cholesterol homeostasis, one of the major unanswered questions is why cells need such an anchor protein. On unloading of the Scap/SREBP cargo in the GA, in theory, the Scap/SREBP complex would be randomly distributed along the GA membrane with a random chance to encounter S1P/S2P for SREBP cleavage. Based on the rule of thermodynamics, the rate of SREBP cleavage would be determined by the duration of SREBP retention in the GA, that is, a longer stay of SREBP in the GA would favour SREBP cleavage. Here comes a question of whether or not the duration for Scap/SREBP to stay in the GA is regulated. Two possibilities may dictate the duration: protein degradation and retrograde traffic. On one hand, Insig-1 in the ER is rapidly ubiquitinated and degraded by proteasome on cholesterol depletion, leading to loss of its anchoring effect for Scap^35^. On the other hand, Insig-1 mediates HMGCR degradation via interaction with gp78 and VCP under cholesterol sufficient condition^36^,^37^,^38^. By analogy, PAQR3 may stabilize Scap/SREBP in the GA and prevent the degradation of Scap and/or SREBP. However, our preliminary data suggest that PAQR3 does not affect the degradation rate of SREBP-2 (Supplementary Fig. 10). Another mechanism that may control the duration of Scap/SREBP complex in the GA is retrograde traffic from the GA to the ER. Whether Golgi-localized Scap/SREBP can be rapidly recycled back to the ER has not been fully investigated. Assuming that the Golgi-localized Scap/SREBP complex can be rapidly and dynamically recycled to the ER, tethering of the complex by PAQR3 would enhance its retention in the GA, leading to reduced retrograde traffic. Our preliminary data are supportive of this hypothesis (Supplementary Fig. 9). Nevertheless, further investigation of these key questions will not only aid in understanding the complexity of Scap/SREBP in the feedback control of cholesterol biosynthesis, but also throw light on the design of new strategies to lower cholesterol in the combat about hypercholesterolemia-mediated cardiovascular diseases.

## Methods

### Cell culture, treatment and transfection

HEK293T, HeLa, HepG2 and Huh7 cell lines were obtained from the American Type Culture Collection, and mouse primary hepatocyte cells were maintained in complete (lipid-loaded) medium containing Dulbecco's modified Eagle's medium (DMEM), 10% foetal bovine serum (FBS), 1,000 U ml^−1^ penicillin and 100 μg ml^−1^ streptomycin. CHO-7 (a clone of CHO-K1 cells selected for growth in LPDS) and SRD-13A (deficient in SCAP derived from CHO-7 cells) were reported and used previously^39^. CHO-7 cells were grown in complete medium containing a 1:1 mixture of Ham's F-12 medium and DMEM (DMEM/F-12), supplemented with 5% (vol/vol) FBS, 100 U ml^−1^ penicillin and 100 μg ml^−1^ streptomycin. SRD-13A cells were grown in complete medium containing a 1:1 mixture of Ham's F-12 medium and DMEM (DMEM/F-12), supplemented with 5% (vol/vol) FBS, 100 U ml^−1^ penicillin and 100 μg ml^−1^ streptomycin, 5 μg ml^−1^ cholesterol, 20 μM sodium oleate and 1 mM mevalonate. All cells were cultured in monolayer at 37 °C in 5% CO_2_. For CHO-7 cells, LD medium contains DMEM/F-12 supplemented with 5% LPDS (Biomedical), 10 μM compactin (Sigma-Aldrich, St Louis, MO, USA) and 50 μM mevalonate (Sigma-Aldrich). For HEK293T, Huh7 and HepG2 cell lines, LD medium contains DMEM supplemented with 5% LPDS, 10 μM compactin and 50 μM mevalonate. All cell lines listed above in LD medium were cultured for 16 h. For SRD-13A cells, LD medium contains DMEM/F-12 supplemented with 5% LPDS, 10 μM compactin, 50 μM mevalonate and 1.5% cyclodextrin, and the cells in this LD medium were cultured for 90 min. 25-HC-replenishing medium consisted of LD medium plus 1 μg ml^−1^ 25-HC (Sigma-Aldrich). Transient transfection was performed using Polyjet (Signagen), lipofectamine 2000 (Invitrogen) or polyethylenime (Sigma-Aldrich) transfection reagents according to the manufacturer's instructions.

### Cholesterol and TG measurements

For measurement of liver TC and TG, 40–50 mg of liver tissue was homogenized in 0.5 ml PBS. About 5 μl of the total homogenates were used for protein quantification. About 0.4 ml homogenates were mixed with 1.6 ml of chloroform/methanol (2:1, v/v) adequately for lipid extraction. After centrifugation at 3,000 r.p.m. for 10 min at room temperature, the lower organic phase was transferred and evaporated to dryness overnight in a chemical hood. The residual liquid was re-suspended in 800 ml of 1% Triton X-100 in absolute ethanol, and then the concentrations of TC or TG were measured using the TC or TG determination kit according to the manufacturer's instructions, respectively (SHENSUO UNF, China). Both TG and TC concentrations were normalized to protein concentrations. For measurement of intercellular TG and TC, the cells cultured in six-well plates were collected in 900 μl PBS. About 60 μl of the total cell suspension were transferred to a new tube and centrifuged at 800*g* for 3 min at 4 °C. Then this portion of the cells were lysed in RIPA buffer (Cell Signaling) and used for protein quantification. In addition, 750 μl of the total cell suspension was used for lipid extraction. After centrifugation at 800*g* for 3 min at 4 °C, the collected cells were mixed with 1 ml of chloroform/methanol (2:1, v/v) adequately on a horizontal shaker for 3 h at room temperature. Then 500 μl NaCl (0.1 M) was added into each reaction tube and mixed thoroughly, followed by centrifugation at 3,700 r.p.m. for 10 min. The following experimental procedures were identical with measurement of liver TC and TG.

### Plasmids

The expression plasmids used in the study were generated according to standard molecular biology techniques. All the plasmids were subjected to sequencing verification. Insig-1 was cloned into p3Xflag-CMV-10 vector (Sigma-Aldrich) by a PCR-based method. The following plasmids were described as previously reported: Myc-tagged PAQR3, green fluorescent protein (GFP)-fused PAQR3 and short hairpin RNA (shRNA) targeted for human PAQR3 (refs 17, 40); Flag-tagged SREBP-2, Flag-tagged SREBP-1a and the SRE-luciferase reporter^34^.

### Western blotting and antibodies

For western blotting, cells were collected and lysed in RIPA buffer, then centrifuged at 12,000*g* for 20 min at 4 °C. The supernatant was harvested and protein concentration was determined using the Bio-Rad protein assay kit (Bio-Rad, Hercules, CA) according to the manufacturer's protocol. Identical amounts of protein were subjected to SDS–PAGE electrophoresis and transferred to pre-activated polyvinylidene fluoride membranes (Millipore). The blotted membranes were blocked with 3% BSA for 1 h at room temperature and incubated overnight with primary antibodies at 4 °C, then with secondary antibodies for 1 h at room temperature. Finally, the bands were visualized using the ECL chemiluminescence kit (Pierce). Antibodies used in immunoblotting were as follows: anti-Myc (9E10, catalogue No. sc-40, diluted 1:1,000), anti-β-actin, anti-S1P (SKI-1, H-300, catalogue No. sc-20757, diluted 1:1,000), anti-SREBP-1 (H-160, catalogue No. sc-8984, diluted 1:1,000 for immunoblotting of mouse liver extracts), anti-Scap (9D5, catalogue No. sc-13553, diluted 1:1,000 for immunoblotting of CHO-7 cells), anti-Scap (C-20, catalogue No. sc-9675, diluted 1:1,000 for immunoblotting of HEK293T cells), anti-PAQR3 antibody (catalogue No. sc-161992, diluted 1:1,000 for immunoblotting of mouse liver extracts and CHO-7 cells) and anti-GFP (catalogue No. sc-8334, diluted 1:1,000) antibodies from Santa Cruz; anti-SREBP-2 (catalogue No. ab30682, diluted 1:1,000 for immunoblotting of HEK293T cells), anti-Insig-1(catalogue No. ab70784, diluted 1:1,000), anti-GM130 (catalogue No. ab52649, diluted 1:1,000) and anti-golgin-97 (catalogue No. ab168287, diluted 1:1,000) antibodies from Abcam; anti-SREBP-2 (catalogue No. 10007663, diluted 1:1,000 for immunoblotting of mouse liver extracts, mouse primary hepatocytes and CHO-7 cells) from Cayman; anti-GAPDH (catalogue No. 21336, diluted 1:1,000) from SignalWay; anti-S2P (MBTPS2, catalogue No. 2157, diluted 1:1,000) from Cell Signaling Technology; anti-PAQR3 antibody (catalogue No. PA5–24654, diluted 1:1,000 for immunoblotting of 293T cell extracts) from Thermo Scientific Pierce; anti-calnexin (catalogue No. C4731, diluted 1:1,000) and anti-Flag (catalogue No. F3165, diluted 1:5,000) antibodies from Sigma-Aldrich. The uncropped scans of all the immunoblotting results are provided in Supplementary Fig. 11.

### Cell fractionation

In the cell fraction assay, 40 10-cm dishes were used for each group. SRD-13A cells were transfected with Myc–Scap or Myc–Scap together with GFP–PAQR3. At 36 h after the transfection, the cells were either subjected to normal complete medium or LD medium containing 5% LPDS, 10 μM compactin, 50 μM mevalonate and 1.5% methyl-beta-cyclodextrin for 90 min, and the cells were then placed on ice and washed twice with PBS and homogenization buffer (10 mM triethanolamine-acetic acid, pH 7.4, 0.25 M sucrose, 1 mM sodium EDTA, protease inhibitor cocktail (Roche)). The washed cells were harvested in 0.8 ml homogenization buffer and homogenized by passing through a 25-gauge needle on a 1 ml syringe for 13 times. After centrifugation at 2,000*g* for 15 min at 4 °C, the post nuclear supernatant was collected and loaded on preformed iodixanol (Sigma-Aldrich) gradients. The discontinuous iodixanol gradients were prepared as 2.65 ml of 24, 19.33, 14.66 and 10%, which were made by diluting a 60% stock of iodixanol with cell suspension medium (0.85% (w/v) NaCl, 10 mM Tricine-NaOH, pH 7.4). After standing at room temperature for 2 h, the gradients were then centrifuged at 37,000 r.p.m. in a SW40Ti rotor (Beckman Instruments) for 4 h. The post nuclear supernatant was loaded on the top of the gradients and centrifuged at 37,000 r.p.m. for another 2 h. Deceleration was performed without brake. A total of 15 fractions (800 μl per fraction) were collected from top to the bottom, and the bottom two fractions containing aggregated material were not analysed further. Aliquots of each fraction were used for further analysis by immunoblotting.

### Co-immunoprecipitation and two-step co-immunoprecipitation

Briefly, for co-immunoprecipitation assay, cells were washed three times and then lysed with lysis buffer (20 mM Tris-HCl, pH 7.5, 137 mM NaCl, 5 mM EDTA, 1% NP-40, 10% glycerol, 50 mM NaF, 1 mM Na_3_VO_4_ and protease inhibitor cocktail) for 30 min at 4 °C. The homogenates were centrifuged for 30 min at 12,000 r.p.m. at 4 °C. About 10% of the supernatant was harvested for western analysis as inputs, while the remaining cell lysate was incubated with indicated antibodies overnight at 4 °C. Protein A/G plus agarose beads (Santa Cruz) were added at 4 °C for another 2 h. The immunoprecipitation beads were washed with lysis buffer for five times, followed by western blotting analysis. For two-step co-immunoprecipitation, 293T cells were transfected with Myc–Scap, Flag-SREBP-2 and GFP–PAQR3. For the control of the first immunoprecipitation, a Scap-expressing plasmid lacking the Myc-tag was used. At 36 h after the transfection, the cells were lysed with lysis buffer, sonicated briefly and centrifuged. The supernatant was incubated with anti-Myc antibody bound to protein A/G-agarose beads for 3 h at 4 °C. The beads were washed with lysis buffer three times, and the Myc–Scap protein complex was eluted with 300 μl of lysis buffer containing 250 mM NaCl and Myc peptide for 3 h at 4 °C. The second immunoprecipitation was performed using 150 μl eluent from the first immunoprecipitation and 350 μl lysis buffer containing 464 mM NaCl and 10 μl anti-GFP antibody or control IgG together with protein A/G-agarose beads.

### Immunofluorescence staining and protein co-localization quantitation

Immunofluorescence staining was performed following conventional procedures. Briefly, cells were grown on coverslips, fixed with 4% paraformaldehyde, permeabilized with 0.1% Triton X-100 in PBS for 10 min. After blocking in 3% BSA for 1 h, the cells were incubated with the primary and corresponding fluorophore-conjugated secondary antibodies. Confocal images were captured with an LSM 510 confocal microscope. The Golgi and ER were determined by immunostaining with antibodies against GM130 and calnexin (Sigma-Aldrich), respectively. Nuclei were stained with Hoechst 33342 (Molecular Probes). The fluorescent secondary antibodies were as follows: Alexa Fluor 488 donkey anti-mouse IgG, Alexa Fluor 488 donkey anti-rabbit IgG and Alexa Fluor 633 goat anti-rabbit IgG were from Life Technologies; Alexa Fluor 546 goat anti-rabbit IgG and Alexa Fluor 546 goat anti-mouse IgG were from Molecular Probes; Cy5-conjugated AffiniPure goat anti-mouse IgG was from Jackson ImmunoResearch Laboratories. All images were taken under the same excitation conditions. The merged pictures were generated by LSM 5 IMAGE Browser (Zeiss, Germany) automatically. Co-localization was quantified by calculating Pearson's correlation coefficient value using the Coloc 2 plugin of ImageJ.

### SRE-luciferase reporter assay

Huh7 cells in 24-well plates were transfected with 0.1 μg SRE-luciferase reporter, 0.075 μg β-galactosidase and 0.5 μg of an empty vector or PAQR3 constructs. At 24 h after the transfection, the cells were subjected to different medium. Cell lysates were measured for luciferase and β-galactosidase activities based on manufacturer's instruction (Promega).

### Animal studies

All animals were maintained and used in accordance with the guidelines of the Institutional Animal Care and Use Committee of the Institute for Nutritional Sciences. All of the experimental procedures were carried out in accordance with the CAS ethics commission with an approval number 2010-AN-8. All mice were housed in colony cages and maintained on a 12 h light/dark cycle. All mice were randomly grouped before any experiments started. The chow diet was from SLAC (Shanghai, China). C57BL/6J mice injected with adenovirus were subjected to a standard fasting/refeeding procedure^41^ or diets with different cholesterol content *ad libitum* for 3 days. Briefly, the fasting group was not allowed to eat from 0700 to 0700 hours for 24 h. The refeeding group was fasted from 1900 to 1900 hours for 24 h, and then refed with high-carbohydrate/low-fat diet (TD 88122; Harlan Teklad) from 1900 to 0700 hours for 12 h. All three groups were killed at 0700 hours. Diets with 0% cholesterol (D12102C) were purchased from Research Diets, Inc. Diets with various concentrations of cholesterol were made by adding 0.02% (w/w), 0.2% (w/w) and 2% (w/w) cholesterol (Sigma-Aldrich) into chow diet. In peptide experiments, C57BL/6J mice were i.p. injected with P6–55 or control peptide (500 μg kg^−1^ per day) dissolved in a carrier solution of 0.5% BSA (in PBS, pH 7.0) for 14 days. The peptides were synthesized by GL Biochem Ltd. (Shanghai, China) with the following sequences: control peptide, RKKRRQRRR; P6–55, RKKRRQRRRLKSAHYIELGSYQYWPVLVPRGIRLYTYEQIPGSLKDNPYI TDGYRAYLP.

### Quantitative real-time PCR (Q-PCR)

Total RNA was prepared from cultured cells or mouse tissues using Trizol reagents (Invitrogen). Equal amounts of RNA were reverse transcribed with a high capacity cDNA reverse transcription kit (Tiangen) and subjected to Q-PCR by using SYBR green mixture (TOYOBO) with ABI 7900 Q-PCR Systems according to the manufacturer's protocol. All reactions were performed in triplicate, and the relative amounts of mRNAs were calculated using the comparative C_T_ method. Primer sequences are listed in Supplementary Table 1.

### Small interfering RNA transfection

The siRNA sequence for hamster PAQR3 ((5′-CCCUACUCUUCACUGGGUU-3′) and the control (5′-UUCUCCGAACGUGUCACGU-3′) were from Genepharma Company (Shanghai, China). The PAQR3 siRNA sequence was selected from four different target sequences and transfected with lipofectamine 2000 (Invitrogen) into SRD-13A cells according to the manufacturer's instructions. At 48 h after the transfection, the cells were collected and the knockdown efficiency was analysed by reverse transcription followed by Q-PCR.

### Mouse primary hepatocytes isolation and adenovirus injection

Primary hepatocytes were isolated from male mice at 8–12 weeks of age. Briefly, after the portal vein of anesthetized mice was cannulated under aseptic conditions, the liver was perfused with 50 ml PBS buffer containing 3.8 mg EGTA and 1% HEPES, followed by digestion with 50 ml of perfusion buffer (Krebs Ringer buffer containing 3.6 mg ml^−1^ glucose, 1 M CaCl2 and 5,000 U of collagenase I (Worthington)) at 37 °C. The liver was then aseptically removed to a sterile 10-cm cell culture dish containing 10 ml cold PBS and 10 ml cold DMEM medium with 10% FBS. After dispersed by aspirating with a large-bore pipette, the isolated mouse hepatocytes were filtrated through a 70-mm cell strainer (Fisher) into a 50-ml centrifuge tube and centrifuged at 500*g* for 5 min at 4 °C. Cells were then re-suspended with 20 ml DMEM medium containing 10% PBS and mixed with cold 12.5 ml Percoll solution adequately (11.25 ml Percoll and 1.25 ml 10 × Hank's balanced salt solution buffer) (Life Technologies). After centrifugation at 1,000*g* for 10 min at 4 °C, the supernatant cells were removed and centrifugated cells were washed with PBS three times and counted. Hepatocytes were plated at 6 × 10^5^ cells per well in DMEM medium containing 10% FBS in collagen-coated six-well plates. Cells were cultured for 8 h before further assays. Adenoviruses of either scrambled sequence or shRNA sequence specific targeted for mouse PAQR3 (5′-GCTTTCCTGTTCTACATTTCC-3′) were generated using the BLOCK-iT Adenoviral RNAi Expression System (Invitrogen) according to the manufacturer's instructions. The viruses were amplified by Genepharma Company. The viruses were administrated via caudal vein injection (5 × 10^8^ p.f.u. viruses per mouse). At 7 days after injection, the mice were subjected to different experiment procedures.

### Statistical analysis

The unpaired and two-tailed Student's *t-*test was used for all the statistical analyses, except for the Pearson's correlation coefficient analysis using the Coloc 2 plugin of ImageJ.

## Additional information

**How to cite this article:** Xu, D. *et al.* PAQR3 modulates cholesterol homeostasis by anchoring Scap/SREBP complex to the Golgi apparatus. *Nat. Commun.* 6:8100 doi: 10.1038/ncomms9100 (2015).

## Supplementary Material

Supplementary Information Supplementary Figures 1-11, Supplementary Table 1.
