# Supplementary material for: PAQR3 modulates cholesterol homeostasis by anchoring Scap/SREBP complex to the Golgi apparatus
Source: Nat Commun. 2015 Aug 27;6:8100. doi: 10.1038/ncomms9100 (PMC4560833; doi:10.1038/ncomms9100)
Supplement: Supplementary Information — Supplementary Figures 1-11, Supplementary Table 1. [file ncomms9100-s1.pdf]

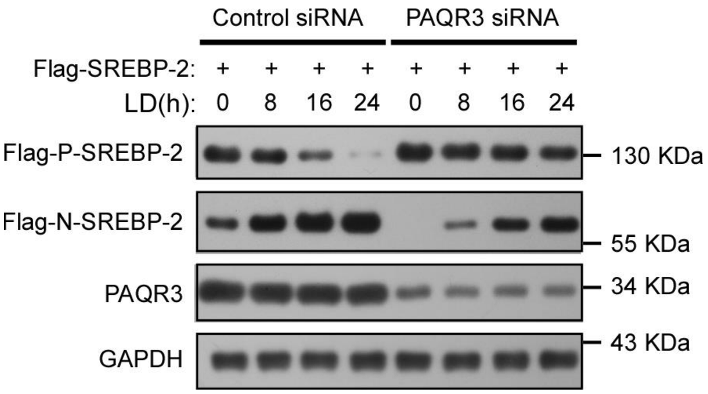

Supplementary Figure 1. PAQR3 knockdown inhibits SREBP-2 processing in CHO-7 cells

CHO-7 cells were transfected with control siRNA or a siRNA targeted for hamster PAQR3. At 24 h after the transfection, the cells were transfected with Flag-tagged SREBP-2 and incubated for 24 h. The cells were then treated with lipid depletion (LD) medium for various times as indicated, following by immunoblotting analysis with the antibodies as indicated.

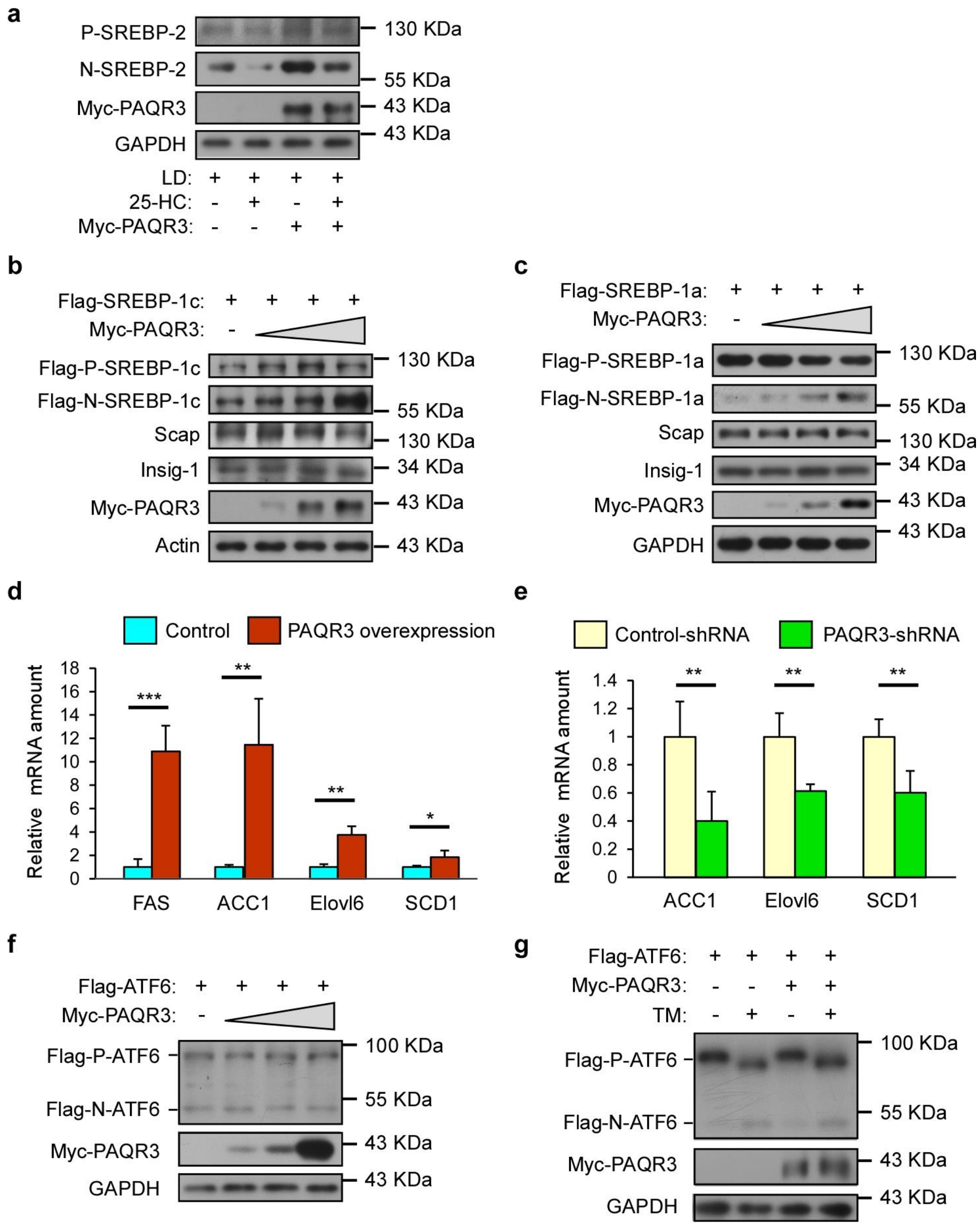

Supplementary Figure 2. Modulation of SREBPs but not ATF-6 processing by PAQR3

(**a**) PAQR3 modulates activation of endogenous SREBP-2. Primary hepatocytes were infected with control or PAQR3-overexpression adenovirus for 24 h and then subjected to

LD medium with or without 25-HC replenishment as indicated. The cell lysate was used in immunoblotting using the antibodies as indicated.

(**b-e**) PAQR3 modulates SREBP-1 activation. Western blot analysis of CHO-7 cells transfected with the plasmids as indicated (**b, c**). Gene expression levels as measured by RT-PCR in HepG2 cells transfected with control plasmid or PAQR3-overexpression plasmid in complete medium (**d**). Gene expression levels as measured by RT-PCR in primary hepatocytes infected with control-shRNA or PAQR3-shRNA adenovirus in complete medium (**e**). All bars show mean ± SD, *p<0.05, **p<0.01 by Student *t*-test.

(**f-g**) PAQR3 does not affect ATF-6 processing. HEK293T cells were transfected with expression plasmids as indicated. At 24 h after transfection, the cells were harvested directly (**f**) or incubated with 4 μM tunicamycin (TM) for 4 h before harvest (**g**). Western blotting was analyzed using the antibodies as indicated. P-ATF6 and N-ATF6 represent precursor and nuclear forms of ATF6 respectively.

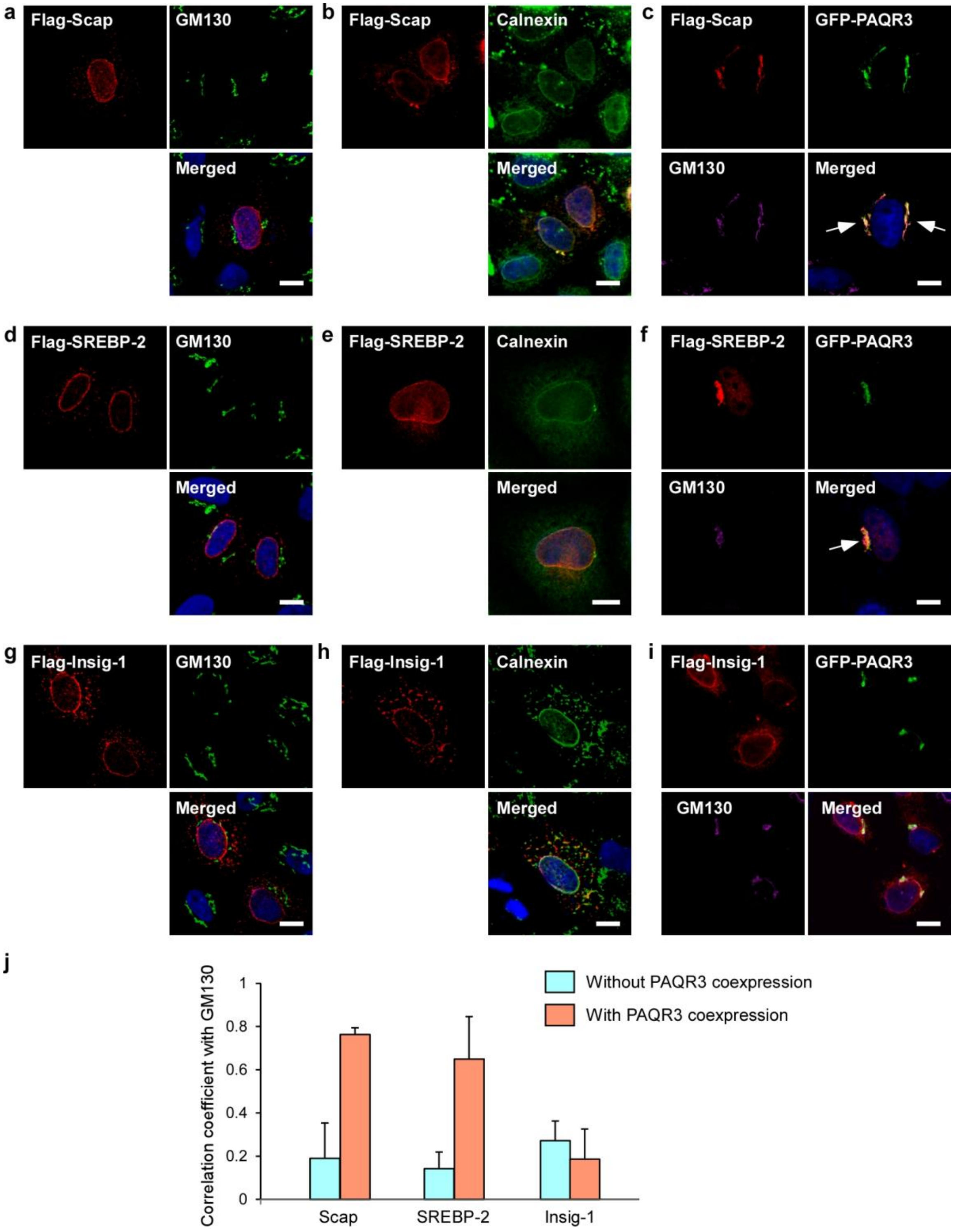

Supplementary Figure 3. PAQR3 tethers Scap and SREBP-2 to the Golgi apparatus

(**a-i**) Overexpression of PAQR3 elevates Golgi localization of Scap and SREBP-2. HeLa cells were transiently transfected with Scap alone (**a, b**), Scap and PAQR3 (**c**), SREBP-2

alone (**d, e**), SREBP-2 and PAQR3 (f), Insig-1 alone (g, h), Insig-1 and PAQR3 (**i**). The arrows indicate co-localization of PAQR3 with Scap (**c**) or SREBP-2(**f**) in the GA. Localization of Scap and SREBP-2 with calnexin or GM130 calculated by ImageJ is shown in **j**. Scale bar: 10 μm.

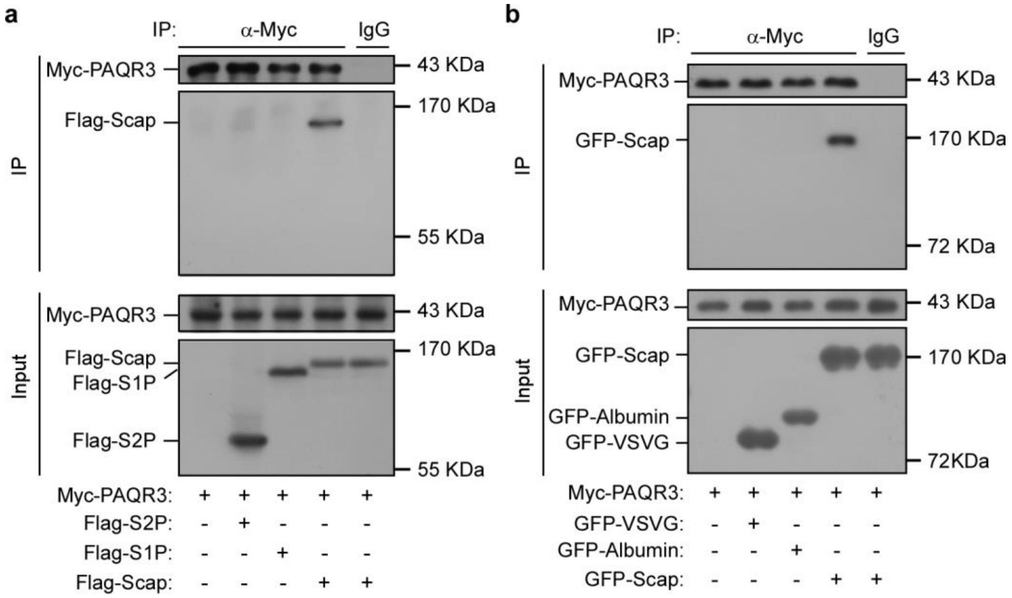

Supplementary Figure 4. PAQR3 does not interact with S1P, S2P, VSVG, and albumin.

(**a, b**) HEK293T cells were transiently transfected with the plasmids as indicated. The cell lysate was used in immunoblotting (IB) and immunoprecipitation (IP) with the antibodies as indicated.

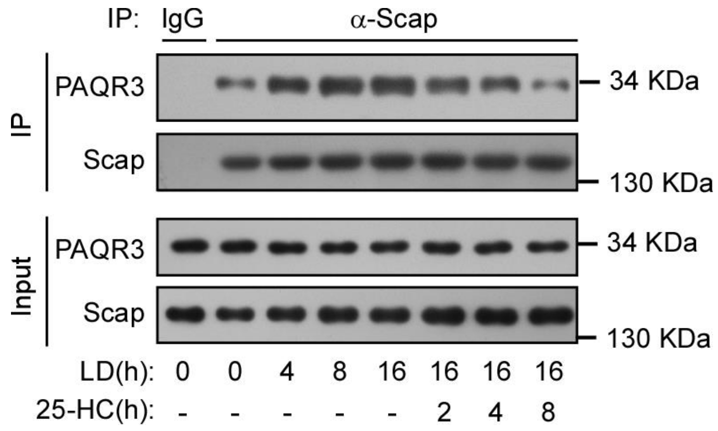

Supplementary Figure 5. Sterols regulate interaction between endogenous Scap and PAQR3

CHO-7 cells were subjected to normal medium, LD medium with or without 25-HC replenishment for different time as indicated. The cell lysate was used in immunoprecipitation (IP) and immunoblotting (IB) to detect endogenous Scap and PAQR3.

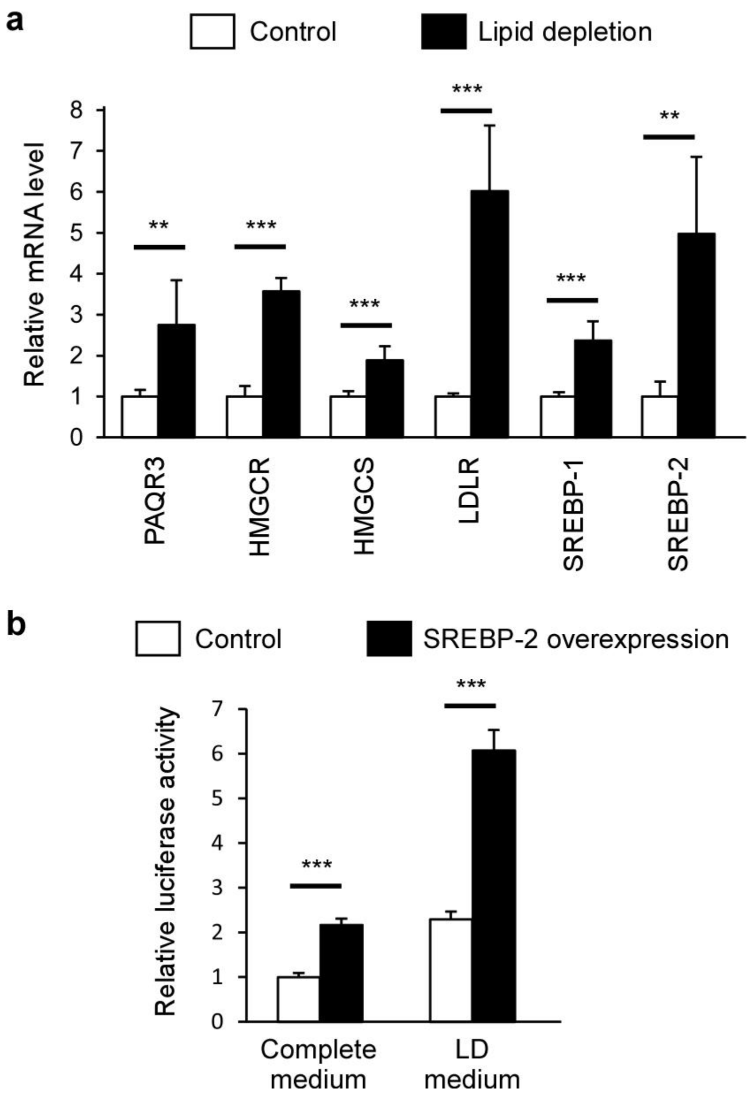

Supplementary Figure 6. The expression of PAQR3 is elevated by lipid depletion

(**a**) Gene expression analysis in HepG2 cells cultured in complete medium or LD medium. (**b**) HEK293T cells were transfected with control plasmid or SREBP-2 expression plasmid together with β-galactosidase and a reporter gene containing a 3-kb human PAQR3 promoter. Luciferase activities measured under lipid-loaded (control) and LD conditions were normalized to β-galactosidase activities. All bars show mean ± SD, $^{**}p<0.01$, $^{***}p<0.001$ by Student *t*-test..

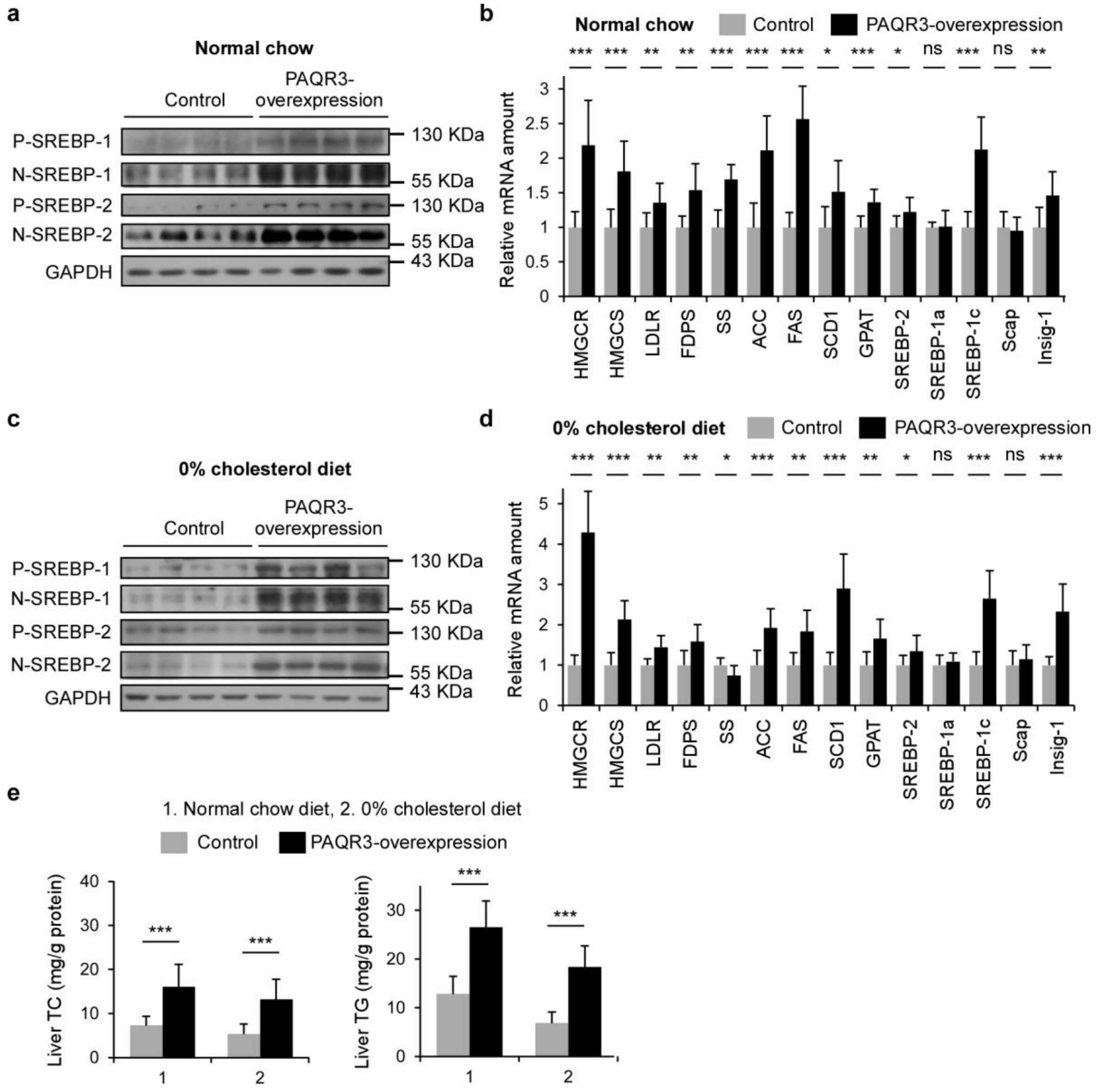

Supplementary Figure 7. Adenovirus-mediated PAQR3 overexpression in the mouse liver promotes SREBP activation and lipid biosynthesis

Seven-week-old C57BL/6J mice were injected with control or PAQR3-overexpression adenovirus by tail vein injection. At 7 days after injection, the mice (n = 8 per group) were subjected to normal chow diet and a diet with 0% cholesterol for 3 days, and then sacrificed for analysis. (**a, c**) Immunoblotting analysis of the mouse liver extracts. (**b, d**) Gene expression profiles as measured by RT-PCR in the mouse livers. (**e**) Liver total

cholesterol (TC) and triglyceride (TG) contents. All bars show mean ± SD, *$p<0.05$, **$p<0.01$, ***$p<0.001$ by Student *t*-test.

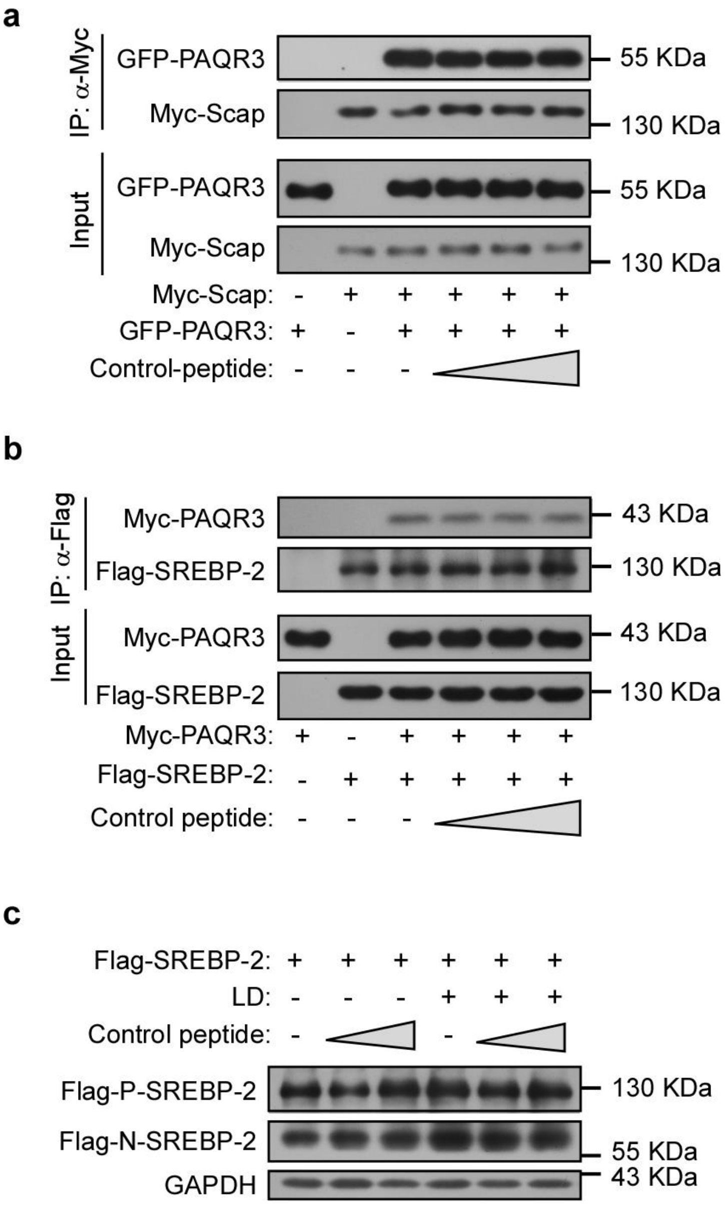

Supplementary Figure 8. A control peptide does not affect SREBP activation

(**a, b**) HEK293T cells were transfected with the plasmids as indicated. At 24 h after transfection, the cells were treated with either PBS or a synthetic control peptide (1, 4 or 20 ng/μl) for 12 h and the cell lysate was used in immunoblotting (IB) and immunoprecipitation (IP) with the indicated antibodies. (**c**) CHO-7 cell was transfected with Flag-tagged SREBP-2. At 6 h after transfection, the control peptide (4 or 20 ng/μl) was added into the complete medium or LD medium for 16 h. The cell lysate was then used in IB with the indicated antibodies.

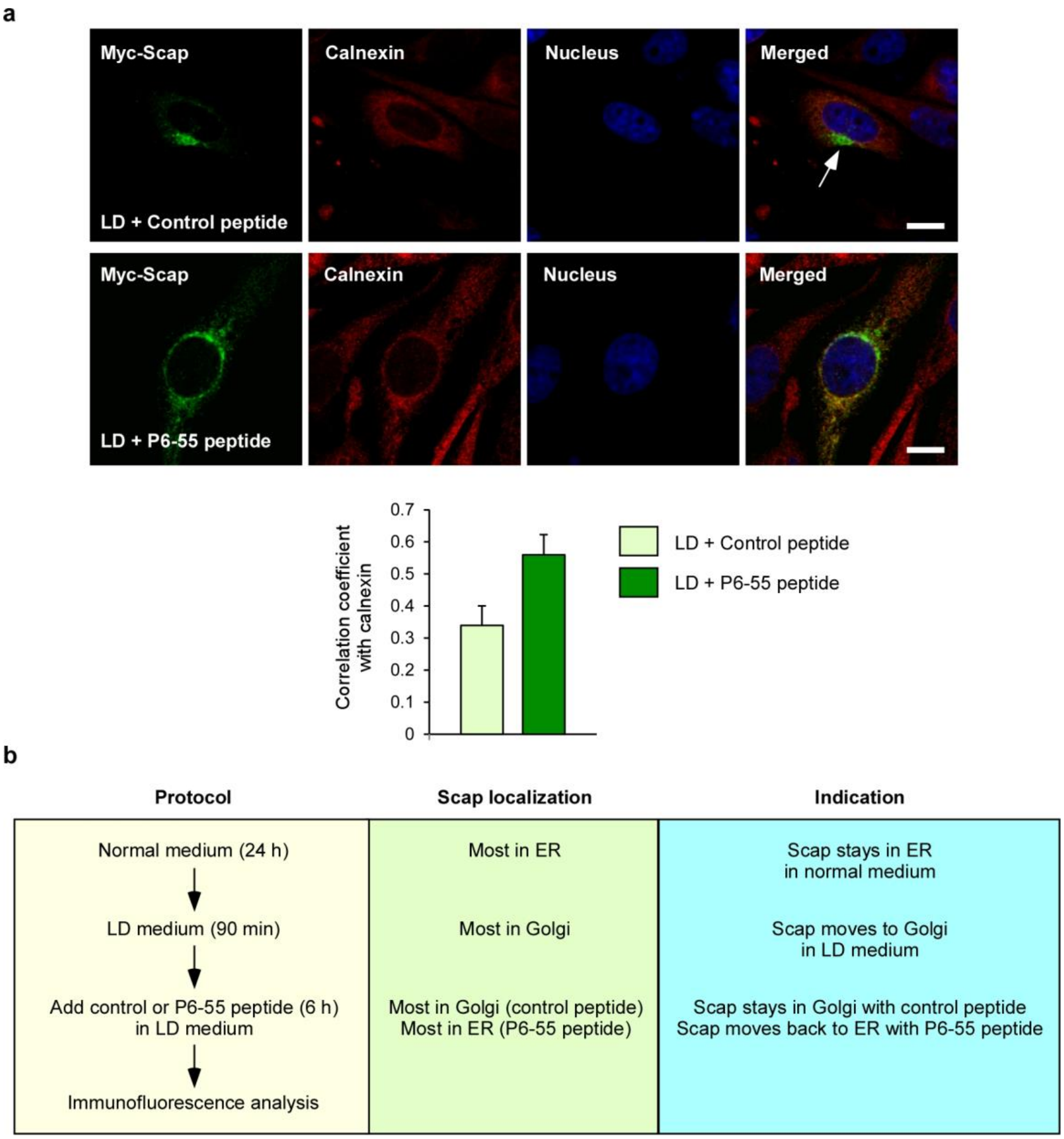

Supplementary Figure 9. PAQR3 affects reverse traffic of Scap from the Golgi apparatus to the ER

(**a**) SRD-13A cells were transfected with Myc-tagged Scap and cultured for 24 h. Then the cells were subjected to lipid depletion (LD) medium containing 5% LPDS, 10 μM compactin, 50 μM mevalonate, 1.5% CDX for 90 min. A control peptide or P6-55 peptide at 20 ng/μl was added in the lipid depletion medium containing 5% LPDS, 10 μM compactin and 50 μM mevalonate. The cells were incubated for another 6 h before immunofluorescence staining. The arrow indicates LD-induced Golgi localization of Scap. Quantitation of the colocalization of Scap with calnexin calculated by ImageJ is

shown in the lower panel. (**b**) Summary of the protocol, Scap localization and indication of this experiment. The data shown in Figure 2b are also used to draw the conclusions. Scale bar: 10 μm.

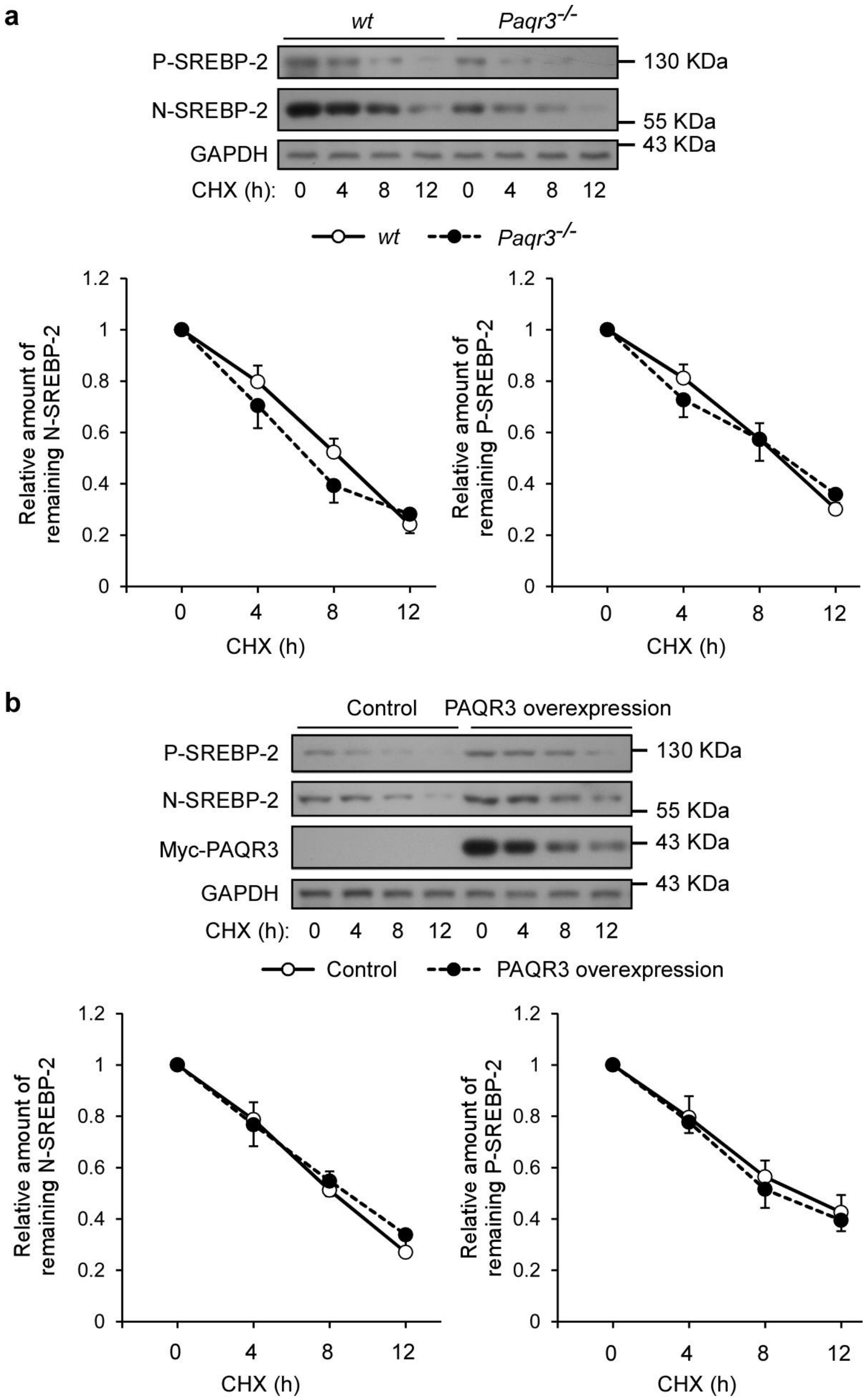

Supplementary Figure 10. The degradation rate of SREBP-2 is not altered by PAQR3

Primary hepatocytes from wild type or Paqr3$^{-/-}$ mice (**a**) or primary hepatocytes infected with control or Myc-PAQR3 overexpressing adenovirus (**b**) were treated with 100 μg/ml

cycloheximide (CHX) for the time as indicated. The immunoblots were quantified and the data were plotted to illustrate the relative amount of remaining SREBP-2 (the data at 0 h was set to 1).

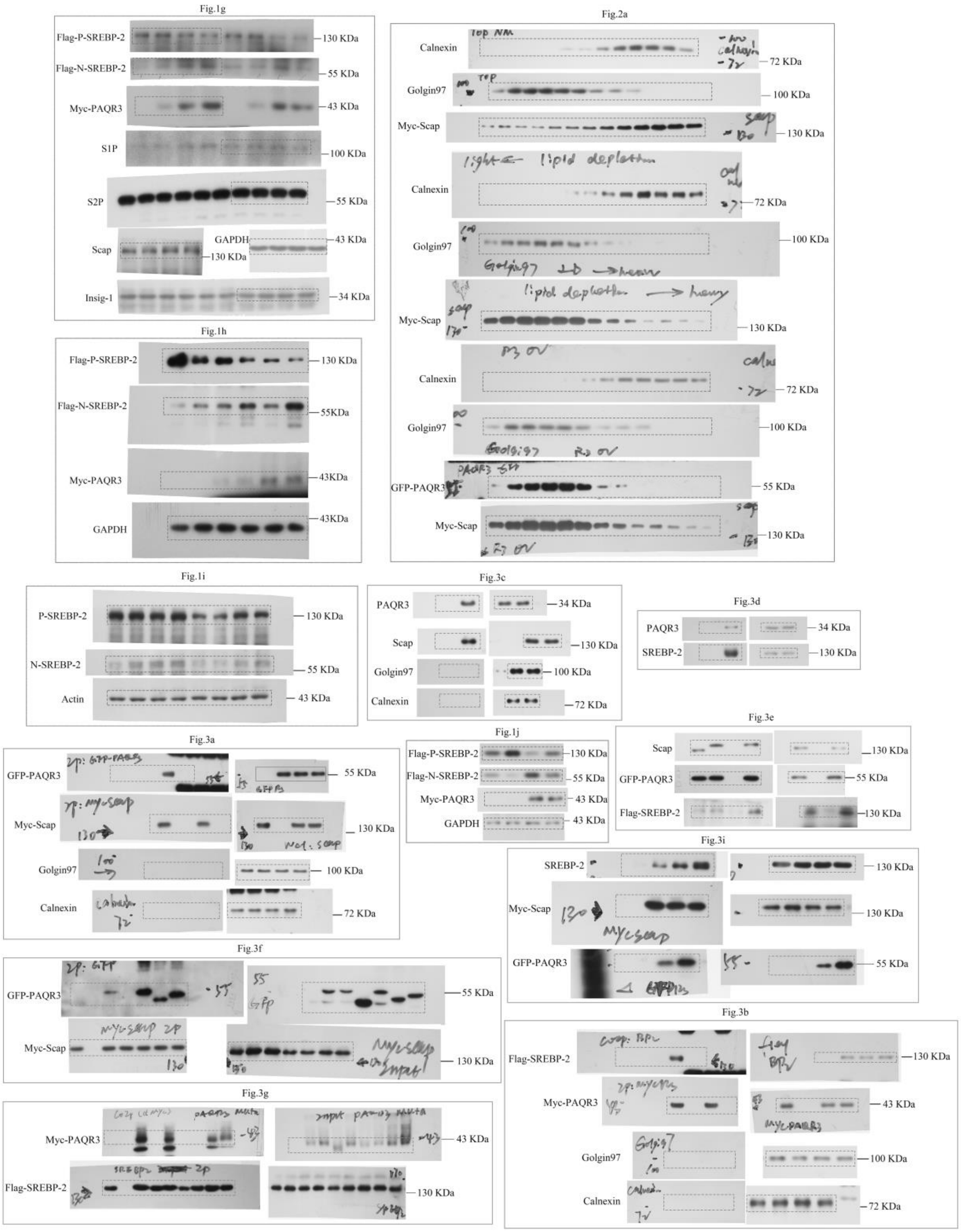

Supplementary Figure 11. Representative original images of immunoblotting results for Figures 1-7.

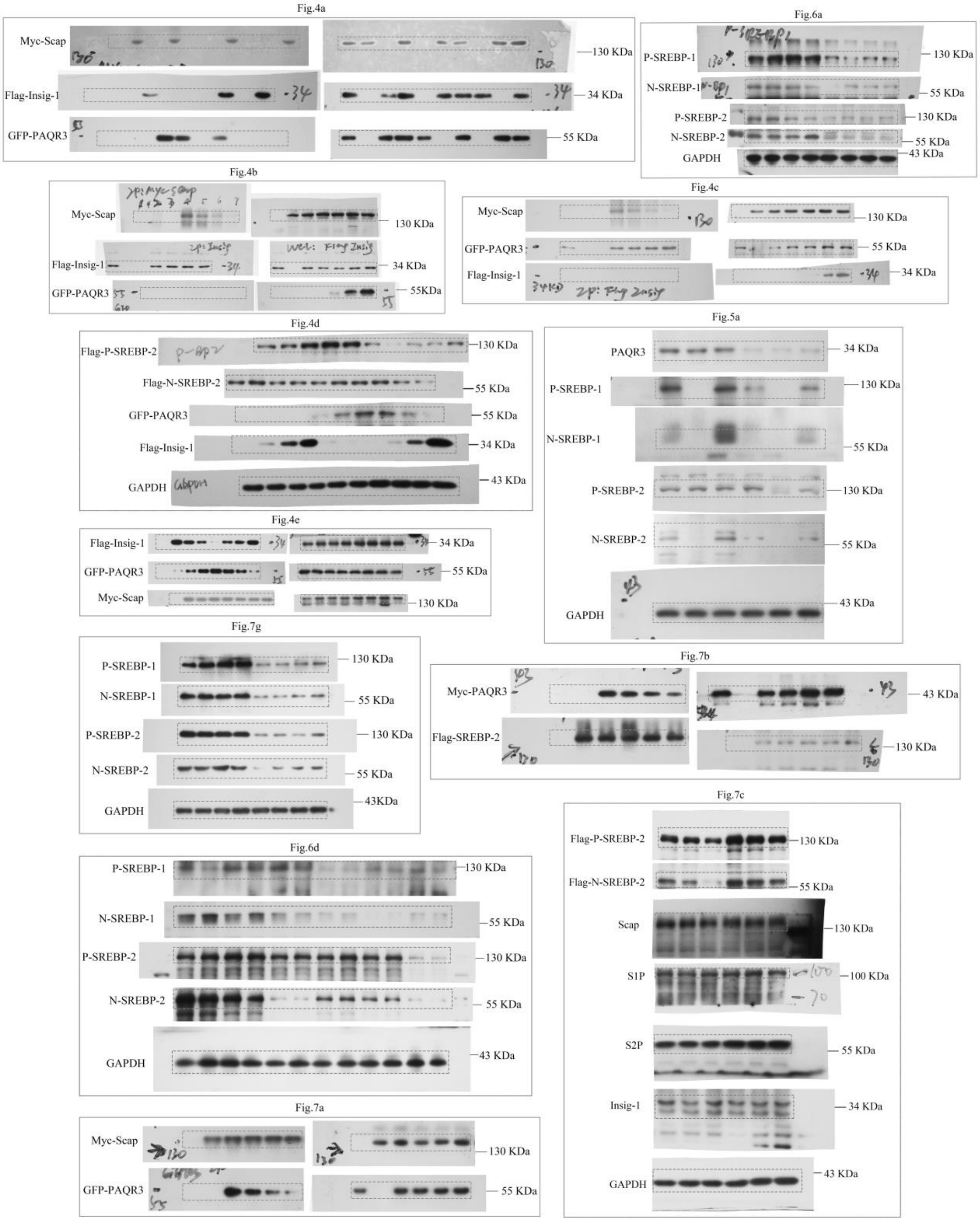

Supplementary Figure 11. Continued

Supplementary Table 1. Primers used for Real-Time PCR			
**Specifies**	**Gene**	**Forward Primer**	**Reverse Primer**
Mus musculus	GAPDH	TGTGTCCGTCGTGGATCTGA	CCTGCTTCACCACCTTCTTGAT
	SREBP-2	GCGTTCTGGAGACCATGGA	ACAAAGTTGCTCTGAAAACAAATCA
	SCAP	ATTTGCTCACCGTGGAGATGTT	GAAGTCATCCAGGCCACTACTAATG
	HMGCS	GCCGTGAACTGGGTCGAA	GCATATATAGCAATGTCTCCTGCAA
	HMGCR	CTTGTGGAATGCCTTGTGATTG	AGCCGAAGCAGCACATGAT
	FDPS	ATGGAGATGGGCGAGTTCTTC	CCGACCTTTCCCGTCACA
	SS	CCAACTCAATGGGTCTGTTCCT	TGGCTTAGCAAAGTCTTCCAACT
	LDLR	AGGCTGTGGGCTCCATAGG	TGCGGTCCAGGGTCATCT
	ACC	TGACAGACTGATCGCAGAGAAAG	TGGAGAGCCCCACACACA
	Fas	GCTGCGGAAACTTCAGGAAAT	AGAGACGTGTCACTCCTGGACTT
	SCD-1	CCGGAGACCCCTTAGATCGA	TAGCCTGTAAAAGATTTCTGCAAACC
	ApoE	GCTGGGTGCAGACGCTTT	TGCCGTCAGTTCTTGTGTGACT
	GPAT	CAACACCATCCCCGACATC	GTGACCTTCGATTATGCGATCA
	IRS-2	GGAGAACCCAGACCCTAAGCTACT	GATGCCTTTGAGGCCTTCAC
	ABCG5	TGGATCCAACACCTCTATGCTAAA	GGCAGGTTTTCTCGATGAACTG
	Insig-1	TCACAGTGACTGAGCTTCAGCA	TCATCTTCATCACACCCAGGAC
	Insig-2a	CCCTCAATGAATGTACTGAAGGATT	TGTGAAGTGAAGCAGACCAATGT
	Insig-2b	CCGGGCAGAGCTCAGGAT	GAAGCAGACCAATGTTTCAATGG
	PEPCK	CCACAGCTGCTGCAGAACA	GAAGGGTCGCATGGCAAA
	SREBP-1a	GGCCGAGATGTGCGAACT	TTGTTGATGAGCTGGAGCATGT
	ACLY	ATATTCATCAGCTTCCTCCC	TCCAAGAAGCCAAATCTTATCCTG
	PAQR3	GATGGCATTGGATTATGCAG	AAGCACGGTGATCAGGTACA
	SREBP-1C	GGAGCCATG GATTGCACATT	GCTTCCAGAGAGGAGCCCAG
	Elovl6	ACAATGGACCTGTCAGCAAA	GTACCAGTGCAGGAAGATCAGT
Homo sapiens	Elovl6	GCACCCGAACTAGGAGATACA	CCCCGGCAACCATGTCTTT
	SCAP	TATCTCGGGCCTTCTACAACC	GGGGCGAGTAATCCTTCACA
	PAQR3	AACCCGTACATCACCGACG	TCTGGACGCACTTGCTGAAG

	HMGCR	TCTGGCAGTCAGTGGGAACTATT	CCTCGTCCTTCGATCCAATTT
	HMGCS	GATGTGGGAATTGTTGCCCTT	ATTGTCTCTGTTCCAACTTCCAG
	LDLR	AACGGTCATTCACCCAGGTC	GGCTGAAGAATAGGAGTTGCC
	SS	CGATAGCTGTGTGCAAAGTAACT	CCATCTGCTGAGTGCTTTCTG
	SREBP-1	CAGCCCCACTTCATCAAGG	ACTGTTGCCAAGATGGTTCCG
	SREBP-2	AACGGTCATTCACCCAGGTC	GGCTGAAGAATAGGAGTTGCC
	β -Actin	GATCATTGCTCCTCCTGAGC	ACTCCTGCTTGCTGATCCAC
	ABCG5	ACCCAAAGCAAGGAACGGGAA	CAGCGTTCAGCATGCCTGTGT
Cricetulus griseus	HMGCR	AGATACTGGAGAGTGCCGAGAAA	TTTGTAGGCTGGGATGTGCTT
	HMGCS	CCTGGGTCACTTCCTTTGAATG	GATCTCAAGGGCAACGATTCC
	LDLR	AGACACATGCGACAGGAATGAG	GACCCACTTGCTGGCGATA
	GAPDH	GCAAGTTCAAAGGCACAGTCAA	CGCTCCTGGAAGATGGTGAT
	Fas	AGCCCCTCAAGTGCACAGTG	TGCCAATGTGTTTTCCCTGA
	ACC	ACACTGGCTGGCTGGACAG	CACACAACTCCCAACATGGTG
	ABCG5	GGTGTCCTCCATGTCTCCTT	GCTGCCTAGGATGCACATTA
